# The Yeast Ess1 Prolyl Isomerase Controls Swi6 and Whi5 Nuclear Localization

**DOI:** 10.1534/g3.113.008763

**Published:** 2014-01-27

**Authors:** David Atencio, Cassandra Barnes, Thomas M. Duncan, Ian M. Willis, Steven D. Hanes

**Affiliations:** * Department of Biochemistry and Molecular Biology, SUNY Upstate Medical University, Syracuse, New York 13210; † Department of Biochemistry, Albert Einstein College of Medicine, Bronx, New York 10461

**Keywords:** proline isomerase, cyclin-dependent kinase sites, nuclear import, biolayer interferometry

## Abstract

The Ess1 prolyl isomerase from *Saccharomyces cerevisiae* and its human ortholog, Pin1, play critical roles in transcription by regulating RNA polymerase II. In human cells, Pin1 also regulates a variety of signaling proteins, and Pin1 misexpression is linked to several human diseases. To gain insight into Ess1/Pin1 function, we carried out a synthetic genetic array screen to identify novel targets of Ess1 in yeast. We identified potential targets of Ess1 in transcription, stress, and cell-cycle pathways. We focused on the cell-cycle regulators Swi6 and Whi5, both of which show highly regulated nucleocytoplasmic shuttling during the cell cycle. Surprisingly, Ess1 did not control their transcription but instead was necessary for their nuclear localization. Ess1 associated with Swi6 and Whi5 *in vivo* and bound directly to peptides corresponding to their nuclear localization sequences *in vitro*. Binding by Ess1 was significant only if the Swi6 and Whi5 peptides were phosphorylated at Ser-Pro motifs, the target sites of cyclin-dependent kinases. On the basis of these results, we propose a model in which Ess1 induces a conformational switch (*cis-trans* isomerization) at phospho-Ser-Pro sites within the nuclear targeting sequences of Swi6 and Whi5. This switch would promote nuclear entry and/or retention during late M and G1 phases and might work by stimulating dephosphorylation at these sites by the Cdc14 phosphatase. This is the first study to identify targets of Ess1 in yeast other than RNA polymerase II.

Posttranslational modification is critical for regulating the localization, stability, and activity of proteins, as well as their ability to form macromolecular complexes (Walsh 2006). Modifications can be covalent, such as phosphorylation, methylation, or ubiquitylation, or noncovalent, such as isomerization of the peptide bond preceding proline residues. The latter is carried out by enzymes known as peptidyl prolyl *cis*/*trans* isomerases (PPIases) (Schmid 1993). PPIases are conserved in all organisms and aid in the folding of newly synthesized proteins (Schiene and Fischer 2000). They also play regulatory roles by inducing conformational changes in mature proteins (Schiene-Fischer *et al.* 2013). Proteins whose activity is regulated by isomerization include those required for nearly every aspect of cellular regulation, including signal transduction, stress response, growth and differentiation, and transcription (Lee *et al.* 2011; Lu *et al.* 2007).

In budding yeast, there are more than a dozen PPIases, most of which have partially redundant, nonessential functions (Arevalo-Rodriguez *et al.* 2004; Dolinski *et al.* 1997). One exception is a PPIase called Ess1, which is essential for growth (Hanes *et al.* 1989; Hani *et al.* 1995). Mutations in Ess1 result in mitotic arrest and nuclear fragmentation (Lu *et al.* 1996; Wu *et al.* 2000). Genetic screens identified a role for Ess1 in transcription (Hani *et al.* 1999; Wu *et al.* 2000). Molecular and biochemical analysis showed that Ess1 binds to the carboxy-terminal domain (CTD) of the largest subunit of RNA polymerase II (RNAPII) and preferentially isomerizes the bond between Ser5 and Pro6 within the heptad repeats (YSPTSPS) (Gemmill *et al.* 2005; Morris *et al.* 1999; Wu *et al.* 2000). Ess1 binding to the CTD depends on phosphorylation of the serine within the Ser-Pro motif (Gemmill *et al.* 2005; Wilcox *et al.* 2004). In turn, Ess1-induced isomerization of the CTD controls the binding and release of transcription cofactors to the RNAPII complex (Singh *et al.* 2009). In *ess1* temperature-sensitive mutant cells, RNAPII function is compromised resulting in defects in initiation, elongation, and termination of mRNAs and small ncRNAs (Ma *et al.* 2012; Singh *et al.* 2009). It is not known whether the arrest phenotype of *ess1* mutants is caused by these transcriptional defects, or whether it results from misregulation of other pathways.

The human ortholog of Ess1, called Pin1 (Lu *et al.* 1996), also has been implicated in RNAPII regulation (Albert *et al.* 1999; Xu *et al.* 2003; Xu and Manley 2007). However, Pin1 also functions in a variety of other pathways and has been shown to bind a wide spectrum of target proteins, including p53, β-catenin, NF-κB, *tau*, and β-APP among others (Lu *et al.* 1999; Pastorino *et al.* 2006; Ryo *et al.* 2001, 2003; Zheng *et al.* 2002). As such, Pin1 has been implicated in many disease states, including cancer, Alzheimer’s disease, autoimmune disease, diabetes, and hematopoietic disorders (Bao *et al.* 2004; Lu 2004; Pulikkan *et al.* 2010; Takahashi *et al.* 2008; Theuerkorn *et al.* 2011). Given the widespread function of Pin1 in mammalian cells, it seemed plausible that Ess1 in yeast may regulate pathways in addition to those involved with RNAPII. Identifying such pathways would be useful for understanding its essential function in yeast, for example, for the future development of antifungal drugs, and to provide a useful model for a deeper understanding of Pin1 function in mammalian cells. Therefore, we set out to identify potential target pathways and proteins by carrying out a large-scale synthetic genetic array (SGA) screen (Tong *et al.* 2001) via a conditional-lethal allele of *ESS1*.

The results identified synthetic genetic interactions between *ESS1* and clusters of genes that fall into distinct categories. As expected, we identified interactions with genes involved in transcription and chromatin modification. However, we also identified clusters of genes involved in stress-response and cell-cycle control. In this paper, we characterize the interactions of *ESS1* with cell-cycle control genes, including *SWI4*, *SWI6*, *WHI5*, and *MBP1*. Surprisingly, Ess1 did not seem to control their transcription. Instead, at least for *SWI6* and *WHI5*, Ess1 controlled the nuclear localization of their protein products. Both proteins have nuclear localization sequences (NLS) containing Ser-Pro motifs that are known to be phosphorylated and are therefore potential substrates for Ess1. Whi5 also has a nuclear export signal (NES) that is regulated by phosphorylation (Taberner *et al.* 2009). Nuclear localization of Swi6p and Whi5p is known to require dephosphorylation of the serine residues at these sites (Harreman *et al.* 2004; Kosugi *et al.* 2009; Taberner *et al.* 2009). Our data suggest a model in which Ess1 binds and isomerizes the phospho-Ser-Pro motifs within the NLSs of Swi6p and Whi5p and the NES of Whi5, and that the resulting conformational changes impact nuclear import/export. One mechanism could be to generate a *cis*/*trans* isomer favorable to the Cdc14 phosphatase, thereby promoting de-phosphorylation and nuclear entry (and nuclear retention). Mislocalization of these cell-cycle regulators in *ess1* mutants may contribute to the cell cycle arrest phenotype previously reported.

## Materials and Methods

### Yeast strains, plasmids, oligonucleotides, media, and growth conditions

*Saccharomyces cerevisiae* strains used are listed in Table 1. YDA692 and YDA695 were created by transforming a *SWI6-GFP* strain (Invitrogen cat. no. 95700) with linearized p500 and p502 to replace the *ESS1^WT^* gene with either *ESS1^WT^*::*natMX4* or *ess1^H164R^*::*natMX4* respectively. This method was also used to introduce the *natMX4*-marked *ESS1^WT^* or *ess1^H164R^* genes into SGA strain Y7092 to generate query strains YDA504 and YDA506 for the SGA experiment. Changes were confirmed by DNA sequencing. Double mutants were generated by crosses and tetrad dissection using deletion strains from the EUROSCARF collection. TAP-tagged strains were created by crosses and tetrad dissection using TAP-tagged strains from the Thermo Scientific collection (cat. no. YSC1177). Plasmids are listed in Table 2 and oligonucleotides in Table S1. Details of their construction are available upon request. Yeast strains were cultured using standard media and growth conditions (Sherman 1991). W303-based strains bearing plasmids were grown in synthetic medium with dextrose and the appropriate amino acid left out, with the addition of 0.5 mg/mL casamino acids and 120 mg/L adenine. For galactose-induction experiments, cells were pregrown to mid-log phase (optical density of a sample measured at a wavelength of 600 nm [OD_600_] ~0.5) in synthetic media SR-ura containing 2% raffinose, collected by centrifugation and resuspended at OD_600_ = 0.4 in SGR-ura containing 1% raffinose and 2% galactose and grown for an additional 3 hr.

**Table 1:** *S. cerevisiae* strains

Strain Name	Description	Source
Y7092	*MATα can1Δ*::*STE2pr-Sp_his5 lyp1Δ his3Δ1 leu2Δ0 ura3Δ0 met15Δ0*	C. Boone
YDA504	*MATα can1Δ*::*STE2pr-Sp_his5 lyp1Δ his3Δ1 leu2Δ0 ura3Δ0 met15Δ0 ESS1*::*natMX4*	This study
YDA506	*MATα can1Δ*::*STE2pr-Sp_his5 lyp1Δ his3Δ1 leu2Δ0 ura3Δ0 met15Δ0 ess1^H164R^*::*natMX4*	This study
YDA579	*ESS1*::*natMX4**^a^*	This study
YDA582	*ess1^H164R^*::*natMX4**^a^*	This study
YDA541	*swi6*Δ *ESS1*::*natMX4**^a^*	This study
YDA545	*swi6*Δ *ess1^H164R^*::*natMX4**^a^*	This study
YDA546	*mbp1*Δ *ESS1*::*natMX4**^a^*	This study
YDA548	*mbp1*Δ *ess1^H164R^*::*natMX4**^a^*	This study
YDA551	*whi3*Δ *ESS1*::*natMX4**^a^*	This study
YDA552	*whi3*Δ *ess1^H164R^*::*natMX4**^a^*	This study
YDA585	*whi5*Δ *ESS1*::*natMX4**^a^*	This study
YDA588	*whi5*Δ *ess1^H164R^*::*natMX4**^a^*	This study
YDA674	*SWI6*-TAP *ESS1*::*natMX4**^a^*	This study
YDA677	*SWI6*-TAP *ess1^H164R^*::*natMX4**^a^*	This study
YDA680	*WHI5*-TAP *ESS1*::*natMX4**^a^*	This study
YDA683	*WHI5*-TAP *ess1^H164R^*::*natMX4**^a^*	This study
YDA692	*MATa his3Δ1 leu2Δ0 met15Δ0 ura3Δ0 SWI6-GFP*::*HIS3 ESS1*::*natMX4*	This study
YDA695	*MATa his3Δ1 leu2Δ0 met15Δ0 ura3Δ0 SWI6-GFP*::*HIS3 ess1H164R*::*natMX4*	This study
YDA699	*ESS1-YFP [HIS]*::*natMX4**^a^*	This study
YDA701	*ess1^H164R^-YFP [HIS]*::*natMX4**^a^*	This study
BY4741 3x-FLAG-Rpb3	*MAT*a *his3*Δ0 *leu2*Δ0 *met15*Δ0 *ura3*Δ0 (C-terminal 3x-FLAG-Rpb3)	(Churchman and Weissman 2011)

a These strains are all derived from BY4741 and/or BY4742 backgrounds and are of genotype *MAT*α *his3*Δ0 *leu2*Δ0 *lys2*Δ0 *met15*Δ0 *ura3*Δ0.

**Table 2:** Plasmids

Plasmid Name	Description	Source
p500.3	PCR2.1 (Invitrogen) with *ESS1* and *natMX4*	D. Samaranayake and S. Hanes, unpublished data
p502.8	PCR2.1 (Invitrogen) with *ess1^H164R^* and *natMX4*	D. Samaranayake and S. Hanes, unpublished data
pAC1202	*AmpR TRP1 CEN SWI6-GFP*	A. Corbett (Harreman *et al.* 2004)
Bd1815	pYES2 *AmpR URA3* 2uM *GALp-SWI6*_1-252_-*GFP*	L. Breeden (Sidorova *et al.* 1995)
pAUA-Whi5	pAUA AmpR *URA CEN ADHp-WHI5-GFP*	H. Yanagawa (Kosugi *et al.* 2009)
pTS395	AmpR *URA3 CEN GALp-GFP*	T. Stearns (Carminati and Stearns 1997)
pCS38	(pRS315) AmpR *LEU2 CEN GFP-NPL3*	C. Guthrie (Gilbert *et al.* 2001)

### SGA screen

An SGA experiment was performed as described previously (Moir *et al.* 2012; Tong and Boone 2007). In brief, query strains (YDA504 and YDA506) were screened against an array of 4292 strains from the viable gene-deletion collection. The deletion array contained duplicate copies of each strain in a 1536 colony per plate format and was screened in duplicate against each query using a Singer Instruments RoToR HDA robot (*i.e.*, four colonies were screened for each double-drug resistant mutant combination). After sporulation, the selection of haploid meiotic progeny and the initial G418 drug selection was performed at the permissive temperature (30°). The final double-drug selection was performed at the semipermissive temperature of 34°. Digital images of the *ESS1*^WT^ control and *ess1*^H164R^ mutant colonies were captured and analyzed using ColonyImager and ColonyScorer software (Tong *et al.* 2007). The scoring software performed colony size normalization on each plate, derived mean colony sizes, SDs, t statistics, and calibrated *P* values (believability score). Initial cutoffs were set at believability scores of > 0.2 and < −0.2, and the gene list was filtered to remove genes linked to the query and other selected markers, *CAN1* and *LYP1*. The resulting gene list (Supporting Information, Table S2) was analyzed for functional categories using the Gene Onology (GO) Slim Mapper tool (http://db.yeastgenome.org/cgi-bin/GO/goSlimMapper.pl). Yeast GO-Slim: Process was selected and all of the GO-Slim Terms were used. The output (Table S3) was converted for use in Cytoscape (Cytoscape.org) to create a network from a subset of these genes.

### RNA isolation, cDNA synthesis, and RT-qPCR

Cells (50 mL) were grown in yeast extract peptone dextrose (YEPD) at 30° to an OD_600_ of 0.5−0.8, washed twice with dH_2_O, and frozen at −80°. RNA was purified as described (Schmitt *et al.* 1990) and stored at −80°. From the RNA preparation (100 μL total), 8 μL was treated with DNase by the addition of 1 μL of 10x buffer and 1 μL (2U) of DNase (Turbo DNA-free Kit; Ambion). The mixture was incubated at 37° for 45 min and then inactivated with 1.1 μL of inactivation solution. We used 1 μL for quantitation with a NanoDrop spectrophotometer (ThermoScientific), and concentrations ranged from 3.2 to 5.8 μg/μL. To control for efficacy of the DNase treatment, a dilution equivalent to what was used for reverse-transcription quantitative polymerase chain reaction (RT-qPCR; 0.05 μg) was subject to PCR using primers for the *SNR6* reference gene (95° 2 min, 40 cycles of 95° 15 sec, 55° 15 sec, and 68° 20 sec). No products were observed on ethidium bromide stained gels. For nocodazole-synchronized cells, cells (250 mL) were grown in YEPD at 30° to an OD_600_ of 0.4. Nocodazole (Sigma-Aldrich; cat. no. M1404) was added to 15 µg/mL and incubated 3 hr at 30°. After release, samples were resuspended at an OD_600_ of 0.4. Samples (50 mL) were collected, washed twice with dH_2_0), and frozen at −80°.

For cDNA synthesis 0.5−1.0 μg of RNA was used in a 10- to 20-μL reaction (respectively) containing random hexamers at 3.75 μM and dNTPs at 500 μM. This was incubated at 65° for 10 min to denature the RNA. 2U-4U of ribonuclease inhibitor (USB), 1U-2U M-MLV reverse transcriptase (USB), 10X buffer (supplied), and water was added to bring the reaction to 1X, followed by incubation at 44° for 1 hr. Then, 0.5 μL of this cDNA and 1 μL of a primer mixture at 10 μM each was added to 6.25 μL of HotStart-IT SYBR-Green qPCR Master Mix (USB) and 4.75 μL of H_2_O. RT-qPCR was performed in an Eppendorf Realplex Mastercycler Epigradient S as follows: 95° 2 min, then 40 cycles of 95° 15 sec, 55° 15 sec, and 68° 20 sec. Before RT-qPCR was performed, all primer sets were tested for efficiency on DNA templates using standard PCR and each reaction resulted in a single prominent band on ethidium bromide-stained gels.

The Comparative C_T_ method (Applied Biosystems) was used for quantitation. The reference gene control was *SNR6* (a Pol III product). For each of the three biological replicates, the C_T_ value for the reference gene was subtracted from the C_T_ value of the query gene, and the three ΔC_T_ values were averaged and a SD (SD_sample_) calculated. This was done for both wild-type and mutant samples. The ΔΔC_T_ values were calculated by subtracting the wild-type ΔC_T_ value from the mutant ΔC_T_ value. The relative (fold) change is calculated as follows: 2^-ΔΔCT^= 2[^ΔCT(WT)-ΔCT(mut)^]. Error bars (SDs) were calculated using the following method (Applied Biosystems): The coefficient of variation (CV) was determined for each sample set (three replicates) by dividing the SD by the mean of the ΔC_T_ values (from above). The plotted SDs (Figure 3) were calculated by the following equation: SD = sqrt[(CV_WT_)^2^ + (CV_mut_)^2^].

### Fluorescence microscopy

Cells were grown to mid-log phase as described previously, and fixed by the addition of a solution containing 0.1 M KPO_4_, pH 7.5, 1.2M sorbitol, and 4% paraformaldehyde. The cells were spun, washed in 0.1M KPO_4_, pH 7.5, 1.2M sorbitol, spun, and resuspended in phosphate-buffered saline. The sample was then brought to 70% ethanol, incubated for 30 min, spun, and resuspended in 0.1M KPO_4_ pH 7.5, 1.2M sorbitol. Immediately before fluorescence microscopy, 4′,6-diamidino-2-phenylindole was added to a final concentration of 0.25 µg/mL and cells spotted onto polylysine-coated slides (slides incubated in 0.01% polylysine for 10 min and allowed to air-dry). The cells were allowed to settle for 10 min before addition of a coverslip. Visualization was done using a Zeiss AxioImager.Z1 microscope with standard filters for green fluorescent protein (GFP) and 4′,6-diamidino-2-phenylindole. Image capture used AxioVision release 4.8 and image processing used AxioVision release 4.9.1.

### Western and CoIP analysis

Western analysis was carried out using standard protocols. Typically, 5 mL of mid-log (OD_600_ ~0.6) cells were collected by centrifugation, washed twice with ice-cold water, resuspended in 20 μL of dH_2_O, and flash frozen on dry ice and stored at −80°. Cell pellets were thawed on ice after adding 4 μL of 12x protease inhibitor consisting of 3 mM phenylmethylsulfonyl fluoride and 8.4 µg/mL Pepstatin A (0.25 mM and 0.7 µg/mL final concentration, respectively). Then, 24 μL of 2× \sodium dodecyl sulfate-polyacrylamide gel electrophoresis (SDS-PAGE) loading buffer with β-mercaptoethanol was added and the mixture and boiled for 10 min. The lysate was mixed, centrifuged, and 10−20 μL of supernatant was then fractionated on SDS-PAGE (7–10% gels). Transfers were done overnight to PVDF membrane (Immobilon-P; Millipore). Protein loading and transfer were monitored using Ponceau S (Sigma-Aldrich) staining of the membrane. Detection was done using ECL-Plus kit (GE) according to the manufacturer’s instructions. Signal was detected with Biomax XAR film (Kodak). Rabbit anti-TAP (Genscript USA Inc., cat. no. 305-035-003) was used at 1:10,000 and rabbit anti-GFP (Invitrogen; cat. no. A6455) was used at 1:25,000. Both were preadsorbed against yeast extract powder made using an acetone preparation (Tom Steven’s laboratory, U. Oregon). Horseradish peroxidase−conjugated goat antirabbit secondary antibodies (Promega; cat. no. W401B) were used at 1:45,000.

Coimmunoprecipitation (CoIP) was performed as follows: One liter of strains YDA674, YDA677, YDA680, and YDA683 or 800 mL of a control strain (BY4741 3x-FLAG-Rpb3) (Churchman and Weissman 2011) were grown in YEPD to an OD_600_ of 1.4. Cells were harvested by filtration, frozen in liquid nitrogen, and lysed as described (Churchman and Weissman 2012). A total of 0.4 mL (from a total of ~4 mL) of grindate was resuspended in 1 mL of binding buffer (20 mM Tris-HCl, pH 8.0; 137 mM NaCl; 1% NP-40; 2 mM ethylenediaminetetraacetic acid; 1 μg/mL leupeptin; 10 μg/mL soybean trypsin inhibitor; 1 μg/mL aprotinin; 0.2 mM benzamidine; 0.1 mM sodium orthovanadate; 5 mM sodium fluoride; 0.25 mM phenylmethylsulfonyl fluoride; and 0.7 μg/mL pepstatin A) and incubated with rocking at 4° for 1 hr. The lysate was pelleted and an aliquot of the supernatant was removed for the whole-cell extract fraction. The remaining supernatant was transferred to a tube containing 200 μL of rabbit IgG-agarose beads (Sigma-Aldrich; A2909) which had been equilibrated with the binding/wash buffer, and incubated with rocking overnight at 4°. The beads were washed 4 times with 1 mL of binding/wash buffer and the supernatants were discarded. 100 μL of 2× SDS protein sample buffer was added to the beads, boiled and spun briefly and the supernatant frozen until use. Samples were separated using SDS-PAGE (15% gel), and the proteins transferred to Immobilon-P as described previously, probed with pre-adsorbed Ess1 antibody (Wu *et al.* 2000) at 1:25,000, and then with True Blot Rabbit HRP (1:2500; Rockland). The blots were developed as previously.

### Kinetic assays of Ess1/peptide binding

Biolayer interferometry (BLI) was measured at 24° with an Octet RED system (FortéBio), essentially as described (Shah and Duncan 2013; Shah *et al.* 2013). Peptides were synthesized with N-terminal biotinylation (NEOBioLab). Phosphoserine residues were incorporated during synthesis and all peptides were purified to >95% purity by high-performance liquid chromatography (NEOBioLab). Each peptide included 4-6 N-terminal residues (typically [Gly-Gly-Ser]_2_) as a flexible linker to facilitate binding of Ess1 protein to the surface-immobilized peptide. BLI assays were carried out in 20 mM Tris-HCl pH 8.0, 1 mg/mL bovine serum albumin (Sigma-Aldrich; A6003-10G). Different peptides were immobilized on separate streptavidin-coated biosensors (FortéBio; cat. no. 18-5019). For each peptide sequence, sensors loaded with phosphorylated or unphosphorylated peptides were assayed in parallel, and the peptide concentrations (50−200 nM) and time of loading were controlled to achieve essentially the same level of peptide signal loaded, regardless of phosphorylation status (*e.g.*, Figure S1). Recombinant *S. cerevisiae* Ess1 protein was purified from *Escherichia coli* as described (Li *et al.* 2005). Ess1 was incubated with peptide-loaded and control (no peptide) biosensors to measure kinetics of Ess1/peptide association, and sensors were moved to buffer alone to measure dissociation kinetics. To correct for low but significant nonspecific binding of Ess1 to sensors, data from a sensor without peptide within the same experiment were subtracted for each concentration of Ess1 used. For direct comparison of Ess1 binding to different peptides (Figure 6), signals were normalized for the levels of sensor-loaded peptide (0.2−0.5 nm) between sequential runs within the experiment. Data were analyzed using FortéBio Data Analysis software (Versions 6.4), and by linear and nonlinear regression in Prism (GraphPad, version 4).

## Results

### SGA screen

A high-copy suppressor screen previously done to identify Ess1-dependent pathways yielded five genes—all related to transcription, and the gene encoding cyclophilin A, another prolyl isomerase (Wu *et al.* 2000). Since that time, additional high-copy and loss-of-function suppressors (all related to RNAPII transcription) have been identified on an individual gene basis (*e.g.*, Krishnamurthy *et al.* 2009; Ma *et al.* 2012; Wilcox *et al.* 2004; Wu *et al.* 2003). However, no systematic screen has been done to identify loss-of-function genetic modifiers of *ESS1* or to identify additional Ess1-dependent pathways. Toward this end, we carried out an SGA analysis to identify mutations that enhance (aggravate) or suppress (alleviate) the growth defect of *ess1* mutant cells (Baryshnikova *et al.* 2010).

Yeast strains bearing the *ess1^H164R^* allele, which encodes a catalytically deficient mutant enzyme, are unable to grow at 37° (Gemmill *et al.* 2005; Wu *et al.* 2000). However, at permissive (25°, 30°) and semipermissive (34°) temperatures, *ess1^H164R^* cells grow at normal or near normal rates. Importantly, molecular defects and genetic interactions have been identified under these conditions (*e.g.*, Ma *et al.* 2012; Singh *et al.* 2009; Wilcox *et al.* 2004; Wu *et al.* 2003). For SGA analysis, we used similar conditions (34°) to score the growth of *ESS1^WT^* and *ess1^H164R^* strains following crosses to a nonessential yeast gene-deletion array. Use of the semipermissive temperature allowed detection of interactions that aggravated growth (synthetic sick or synthetic lethal) as well as interactions that alleviated the mild growth defect (suppression or synthetic rescue).

Approximately 348 potential aggravating interactions and 81 potential alleviating interactions were identified (Table S2). The corresponding genes were categorized by biological process using the Saccharomyces Genome Database Gene Ontology Slim Mapper (Table S3). Among the major categories are (1) response to chemical stimulus, (2) transcription regulation, (3) ion transport, (4) signaling, and (5) regulation of cell cycle (Table 3). Ironically, some of the GO categories in which Ess1 is known to be important (RNAP II transcription, chromatin, mRNA processing) were not significantly enriched in our dataset (Table 3 and data not shown). This could reflect the fact that for some processes such as transcription, Ess1 function may impact genes that are essential and therefore not represented in the screen. In addition, the GO analysis indicates that loss of Ess1 activity has broad consequences in the cell resulting in a functionally diverse set of genetic interactions. Certain GO categories were modestly enriched over a range of SGA score cutoffs including regulation of cell cycle and cofactor metabolic process (Table 3).

**Table 3:** Summary of SGA results by GO analysis

GO Process Slim Term	No. Hits at SGA Score >|0.2|*^a^*	*P* Value at SGA Score >|0.2|*^b^*	*P* Value at SGA Score >|0.25|*^c^*	*P* Value at SGA Score >|0.3|*^c^*	*P* Value at SGA Score >|0.4|*^c^*
Regulation of cell cycle	18	0.042	0.032	0.019	0.030
Cofactor metabolic process	15	0.016	0.007	0.029	0.040
Cellular amino acid metabolic process	20	0.062	0.064	0.060	0.055
Ion transport	22	0.023	0.035	0.047	0.062
Response to chemical stimulus	31	0.033	0.045	0.046	0.068
Signaling	21	0.031	0.065	0.082	Not enriched
Protein targeting	20	0.088	0.077	0.072	Not enriched
Carbohydrate metabolic process	21	0.073	0.058	Not enriched	Not enriched
Transcription from RNAPII promoter	23	Not enriched	Not enriched	Not enriched	Not enriched

Not enriched, *P* > 0.1. SGA, synthetic genetic array; GO, Gene Ontology; RNAPII, RNA polymerase II.

a From Table S2.

b Hypergoemetric distributions were calculated from Table S3.

c Hypergoemetric distributions were calculated from Saccharomyces Genome Database GO Slim Term analysis using gene lists from Table S2 at the indicated cutoff score.

**Figure 1:**
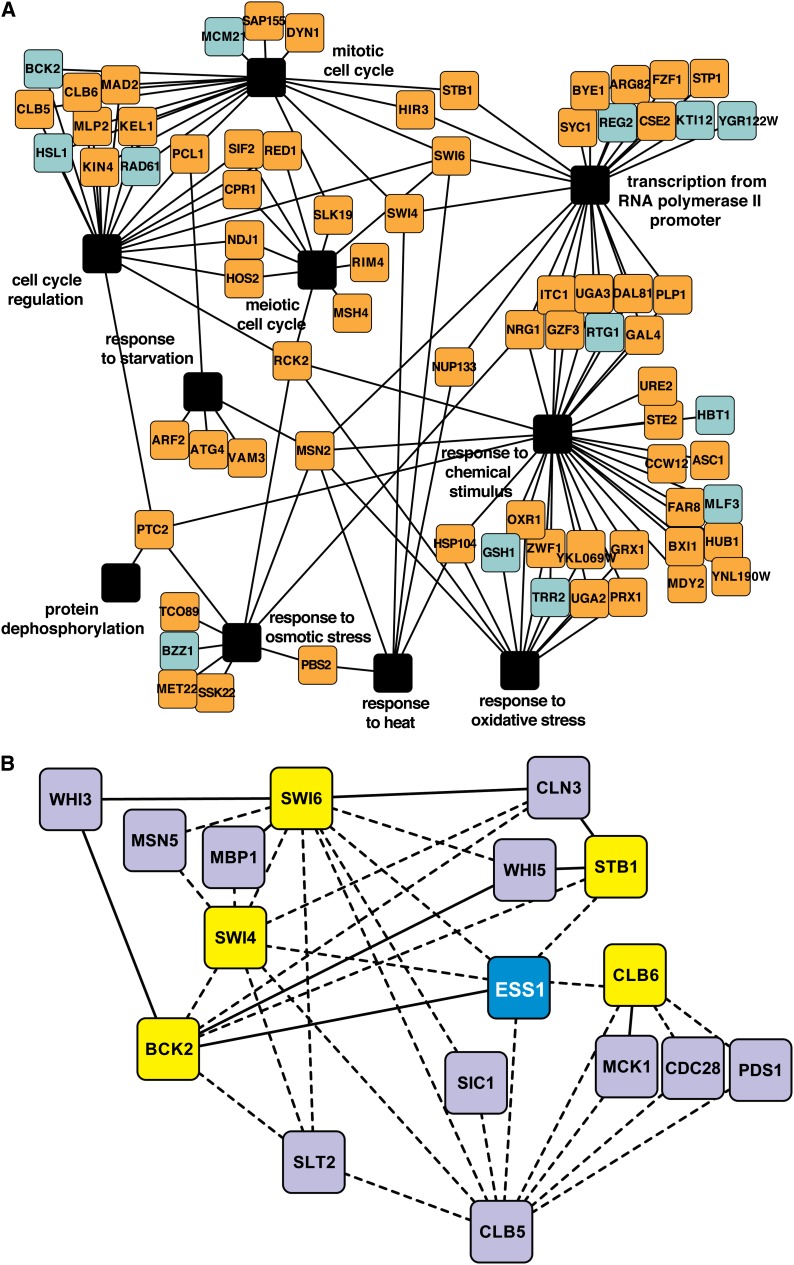
Results of a synthetic genetic array (SGA) screen. (A) Network of genes that interact with *ESS1* as revealed by SGA analysis. All of the genes have SGA scores (calibrated *P* values) > |0.2| (Table S2). Only selected functional categories are shown. Colors indicate which interactions are aggravating (orange) or alleviating (light blue). Functional nodes are in black. (B) Network of *ESS1* interactions with selected cell-cycle genes. This includes genes that interacted directly with *ESS1* (yellow) and secondary interactions of genes that interact with *ESS1*-interacting genes (purple). Line types indicate which interactions are aggravating (dashed) or alleviating (solid). An interaction between *ESS1* and *WHI3* was initially in the SGA screen (Table S2). However, subsequent analysis (see Figure 2) did not confirm the interaction. The map was modified to remove this interaction.

To visualize the results with respect to major GO functional categories of interest, we constructed a network map of a subset of genes that interact with *ESS1* (Figure 1A). As noted previously, a number of interactions were found with genes involved in RNAPII transcription, including one (*BYE1*) that has been previously characterized (Wu *et al.* 2003; Kinkelin *et al.* 2013). A subset of these genes was also associated with the response to chemical stimulus. Genetic interactions involving this category are consistent with previous studies showing elevated sensitivity of *ess1^H164R^* mutants to a variety of stress and chemical insults (Gemmill *et al.* 2005). Interactions between *ESS1* and genes involved in the cell cycle are also highlighted.

We expanded the cell-cycle portion of this network to include secondary interactions, that is, genes that have interactions (described in *Saccharomyces* Genome Database) with more than one of the *ESS1*-interacting genes in our dataset (Figure 1B). We were especially interested in *ESS1* interactions with cell-cycle genes because to date, there is no adequate explanation for why *ess1* mutant cells arrest with a cell cycle−specific terminal phenotype (late M-phase), or what triggers nuclear fragmentation in *S. cerevisiae ess1* mutants (Wu *et al.* 2000). Moreover, the essential function of Ess1 has never been definitively demonstrated; for example, some of the transcriptional defects have been shown to be nonessential because suppressors that rescued growth did not correct the specific transcription defect (Wu *et al.* 2003).

Among the genes that interacted with *ESS1* are *SWI4* and *SWI6*, components of the SBF transcriptional activator complex, which drives cell cycle entry in late G_1_ phase and is repressed during M and early G_1_ phase through a direct physical interaction with Whi5 (reviewed in Wittenberg and Reed 2005). *WHI3*, which also interacted with *ESS1*, encodes a protein that helps restrict the activity of Cdc28 by holding it in the cytoplasm until G_1_/Start (Wang *et al.* 2004), after which time Cdc28 enters the nucleus and phosphorylates Whi5, causing it to exit the nucleus. In the absence of Whi5, SBF (Swi4/Swi6) is derepressed and stimulates transcription of cyclin genes (*CLN1* and *CLN2*) and genes required for spindle pole body duplication. Later in G_1_, Swi6 functions with Mbp1 (MBF-complex) to activate additional cyclins (*CLB5* and *CLB6*) as well as genes required for DNA synthesis, thus promoting entry into S-phase. The observed interactions suggest a possible role for the Ess1 isomerase in regulation of cell-cycle proteins.

### An *ess1* mutant interacts genetically with *swi4*Δ, *swi6*Δ, *whi5*Δ, and *mbp1*Δ

**Figure 2:**
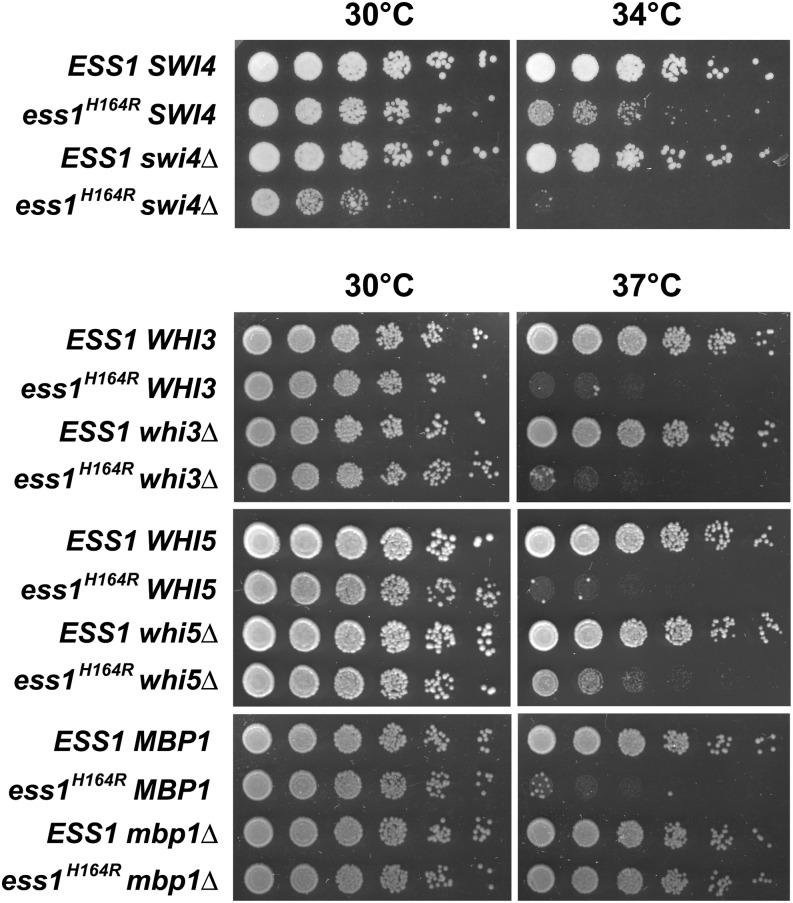
Genetic interactions between *ESS1* and cell-cycle genes. Serial dilutions (1:5, starting with cells at OD_600_ 0.5) of wild-type or the indicated mutants were grown at 30°, 34°, or 37° on yeast extract peptone dextrose for 3 d. *ess1^H164R^ swi4*Δ double mutants show synthetic growth defects, whereas *ess1^H164R^ whi5*Δ and *ess1^H164R^ mpb1*Δ double mutants show synthetic growth rescue. Little or no effect was seen on growth of *ess1^H164R^ whi3*Δ double mutants (*vs. ess1^H164R^* alone). *ess1^H164R^ swi6*Δ double mutants were synthetic lethal (Table 4).

Using standard genetic crosses, we attempted to generate double mutants with *swi4*Δ, *swi6*Δ, *whi3*Δ, mutations for which we observed direct genetic interactions. We also included two genes, *whi5*Δ and *mbp1*Δ, that showed indirect interactions with *ess1^H164R^* and whose functions are closely related. Double mutants were obtained in four of the five crosses (Table 4). The exception, *swi6*Δ, appears to be synthetic lethal with *ess1^H164R^*, even at the permissive temperature (30°). Of the 38 *ess1^H164R^swi6*Δ double mutants expected from our tetrad analysis (Table 4), only four were obtained, presumably due to the presence of background suppressors, and these grew very poorly (data not shown). For the remaining double mutants, growth was monitored relative to the single mutant strains (Figure 2). We found that *ess1^H164R^swi4*Δ double mutants grew very slowly (synthetic sick) at the semipermissive temperature (34°). In contrast, the *ess1^H164R^whi5*Δ and *ess1^H164R^mbp1*Δ double mutants grew modestly better, and significantly better, respectively, at the restrictive temperature (37°) than the *ess1^H164R^* single mutant. Thus, deletion of *SWI4* aggravates, and deletions of *WHI5* and *MBP1* alleviate, the growth defects of the Ess1 mutant. Tetrad analysis followed by growth at 25°, 30°, 34°, or 37° was unable to confirm a genetic interaction between *ESS1* and *WHI3* at (Figure 2 and data not shown).

**Table 4:** Segregation analysis of double mutants

Cross (Relevant Genotypes)	Total Tetrads	No. Viable Spores				
		4	3	2	1	0
*ESS1* x *swi4*Δ	22	5	8	7	2	0
e*ss1^H164R^* x *swi4*Δ	23	11	8	4	0	0
*ESS1* x *swi6*Δ	31	15	14	2	0	0
e*ss1^H164R^* x *swi6*Δ	34	3	20	4	6	1
*ESS1* x *whi3*Δ	24	17	5	2	0	0
e*ss1^H164R^* x *whi3*Δ	24	16	6	1	1	0
*ESS1* x *whi5*Δ	21	14	2	5	0	0
e*ss1^H164R^* x *whi5*Δ	24	20	3	1	0	0
*ESS1* x *mbp1*Δ	23	5	16	2	0	0
e*ss1^H164R^* x *mbp1*Δ	23	10	11	2	0	0

Strains used in crosses: *ESS1* = YDA579; *ess1^H164R^* = YDA582; deletion mutants are from EUROSCARF collection. Frequency in which the dead spore is a double mutant (deduced) in tetrads with 3:1 segregation for growth: *ess1^H164R^ swi*4Δ = 6/8; *ess1^H164R^ swi*6Δ = 16/20; *ess1^H164R^ whi3*Δ = 2/6; *ess1^H164R^ whi5*Δ = 0/3; and *ess1^H164R^ mbp1*Δ = 3/11.

**Figure 3:**
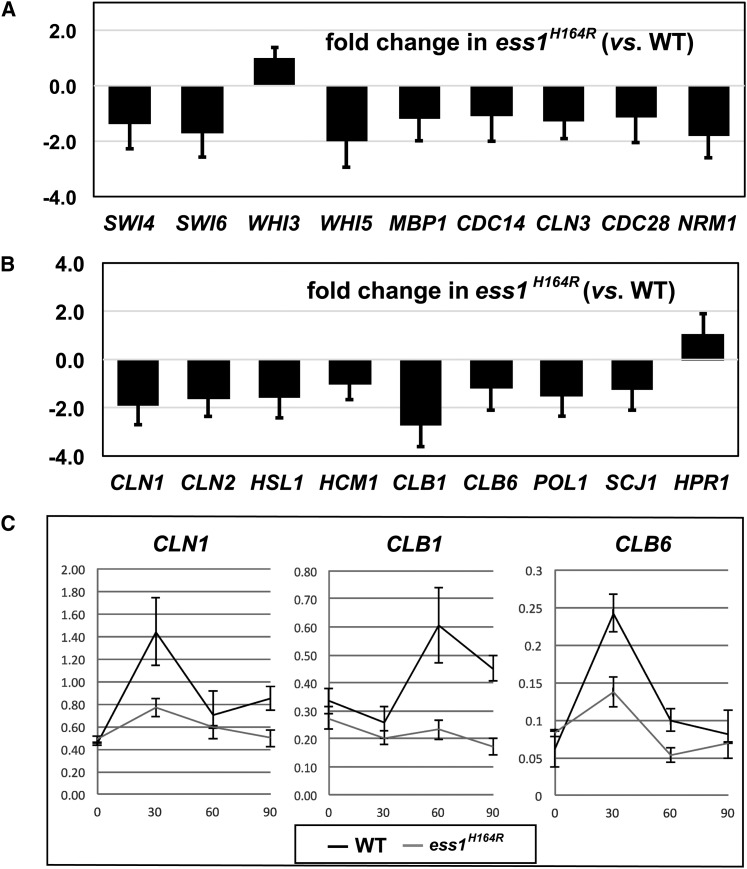
Expression of cell-cycle transcription regulators (A) and cell-cycle target genes (B, C) in *ess1^H164R^* mutants. Total RNA was harvested from wild-type and *ess1^H164R^* cells, reverse transcribed, and quantitated with the use of reverse-transcription polymerase chain reaction (see the *Materials and Methods*). Data are presented as fold-change in expression of the indicated gene in *ess1^H164R^* mutant cells relative to the wild type. Three biological replicates were used for each strain and error bars are the SD from the mean. For samples in (C), cells were grown in yeast extract peptone dextrose at 30°, arrested for 3.0 hr with 15 μg/mL nocodazole, released, and grown for the indicated times prior to harvesting for RNA analysis.

Given that Ess1 is known to be important for RNAPII function, we wondered whether the observed genetic interactions could result from a general defect in the transcription of cell-cycle regulatory genes in the *ess1^H164R^* mutant. To test this, we used RT-qPCR to monitor mRNA levels for the following set of genes, *SWI4*, *SWI6*, *WHI3*, *WHI5*, *MBP1*, *CDC14*, *CLN3*, *CDC28*, and *NRM1*. These genes all encode cell-cycle transcription regulators or kinases/phosphatases that regulate their activity. In no case did we observe a significant change (>2 fold) in expression of these genes in wild-type *vs. ess1^H164R^* cells (Figure 3A).

We next considered that the genetic interactions might reflect a more direct role of Ess1 in controlling the activity of cell-cycle regulators rather than their expression. Indeed, many of these proteins are targets of cyclin-dependent kinases that phosphorylate Ser-Pro motifs, creating potential substrate sites for Ess1 (phospho-Ser-Pro). If Ess1 affects the activity (or localization) of Swi6 and Whi5 proteins, then we might expect to see changes in expression of their target genes in *ess1* mutant cells. Quantitation of mRNA levels from asynchronous cell cultures did not reveal significant effects on the expression of nine target genes with the possible exception of *CLB1*, which was reduced >2-fold. However, when cells were arrested with nocodazole and released, differences were detected in the kinetics of expression of each of the genes examined (Figure 3C). The most significant effects were on *CLN1*, *CLB1*, and *CLB6*, which failed to induce in *ess1^H164R^* cells as strongly as in the wild type. Lesser effects were seen on the induction of *CLN2* and *POL1* expression (data not shown). *CLN1*, *CLN2*, *CLB1*, and *CLB6* are targets of Swi6 (SBF), and *POL1* is a target of Mbp1 (Iyer *et al.* 2001).

### Nuclear localization of Swi6p and Whi5p is defective in *ess1* mutants

**Figure 4:**
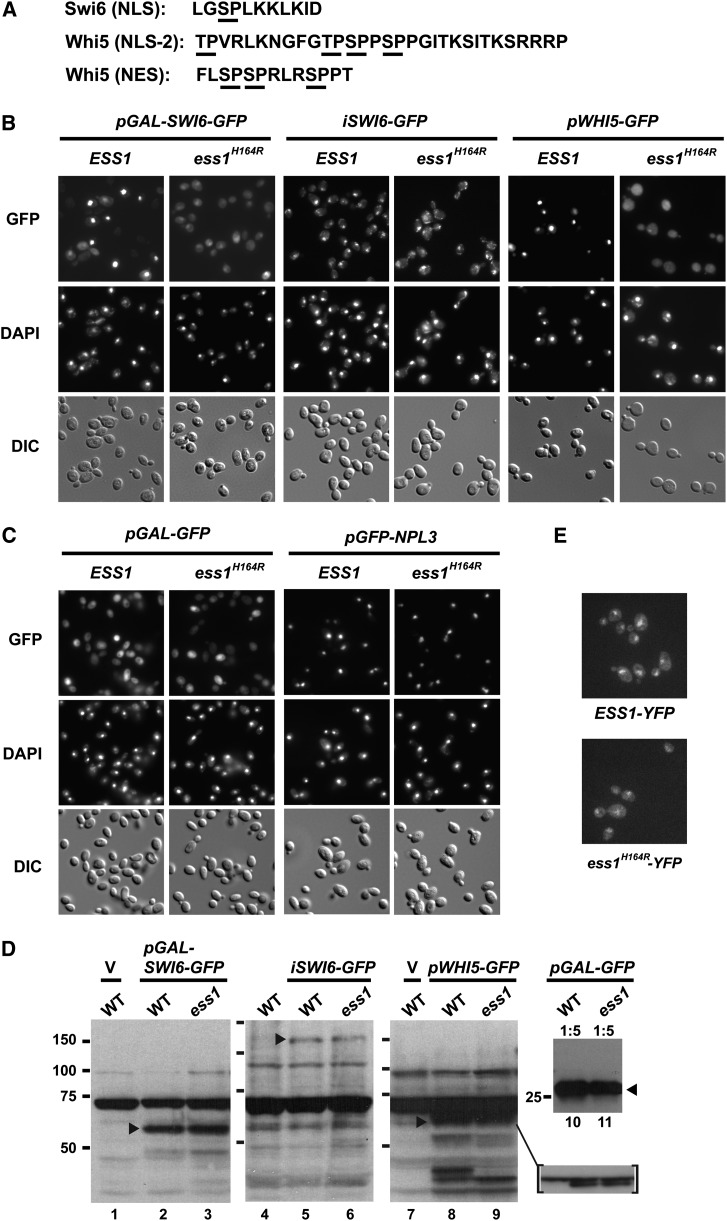
Reduced nuclear localization of Swi6 and Whi5 in *ess1^H164R^* mutant cells. (A) NLS in Swi6 (158−168), Whi5 (47−77) (Kosugi *et al.* 2009; Sidorova *et al.* 1995), and portion (152−164) of a nuclear export sequence in Whi5 (Taberner *et al.* 2009). Sites that are known to be phosphorylated are underlined. These correspond to potential substrate sites for Ess1 prolyl isomerization (*i.e.*, phospho-Ser-Pro, phospho-Thr-Pro). (B) Localization of Swi6-GFP and Whi5-GFP fusion proteins in wild-type and *ess1^H164R^* cells. *pGAL-SWI6* (Bd1815) drives expression of a truncated Swi6 protein (aa1-252) fused to *GFP* under control of the *GAL1* promoter. *iSWI6-GFP* is an integrated fusion gene that expresses full-length Swi6-GFP driven by the native *SWI6* promoter. *pWHI5-GFP* (pAUA-Whi5) expresses full-length Whi5-GFP driven from the *ADH1* promoter. (C) Controls for an unlocalized protein (GFP alone) and a nuclear-localized protein (Npl3). *pGAL-GFP* (pTS395) expresses GFP from the *GAL1* promoter. *pGFP-NPL3* (pCS-38) expresses full-length Npl3 from the native *NPL3* promoter with a GFP inserted after the start codon in the amino terminus (Gilbert *et al.* 2001). For (B) and (C), cells were grown in selective media except for *iSWI6-GFP* cells, which were grown in yeast extract peptone dextrose. Cells with *GAL1*-inducible constructs were induced with galactose as described (*Materials and Methods*). (D) Western analysis of strains expressing Swi6 and Whi5 GFP-fusion proteins. Strains and growth conditions were the same as in (B) and (C) above. Cells transformed with vector only (V) were included as controls. Samples (lanes 1−11) were resolved on 10% SDS gels, the inset samples were resolved on a 7.5% gel. Lanes 4−6 have 2× the amount of extract loaded relative to lanes 1−3 and 7−9. Lanes 10 and 11 have 1/5× loaded. The inset lanes had 1/2× loaded. Blots were probed with rabbit anti-GFP antibodies at a 1:5000 dilution (lanes 1−3, 7−9, 10, 11), or at 1:25,000 (lanes 4−6, inset). Secondary antibodies were diluted to 1:25,000 (lanes 1−3, 7−9, 10, 11) or at 1:45,000 (lanes 4-6, inset). Reactive bands are indicated by arrowheads and migrate at approximately their predicted molecular weights: Swi6_1-252_GFP (55,274 kD), Swi6-GFP (117,491 kD), Whi5-EGFP (59,741 kD), GFP (26,950 kD). (E) Localization of wild-type and mutant Ess1 using strains bearing integrated *ESS1-YFP* or *ess1^H164R^-YFP* fusion genes. Cells were grown to mid-log phase in complete synthetic media at 30C. Images in (E) were captured using an UltraView VoX spinningdisk confocal system (Perkin Elmer Inc.), installed on a Nikon TiE microscope with a Hamamatsu CCD Camera using Velocity software. GFP, green fluorescent protein.

Swi6 contains a Ser-Pro site that resides within its NLS (Figure 4A). Phosphoregulation of this site is important for nuclear-cytoplasmic shuttling of Swi6 (Harreman *et al.* 2004; Sidorova *et al.* 1995; Taberner *et al.* 2009; Wagner *et al.* 2009). Phosphorylated Swi6 is retained in the cytoplasm during M phase. During G_1_, the Ser-Pro site within the NLS is dephosphorylated by the Cdc14 phosphatase allowing Swi6 to enter the nucleus and bind DNA (Geymonat *et al.* 2004). A similar mechanism may also control the nuclear localization of Whi5 (Taberner *et al.* 2009; Wagner *et al.* 2009), which contains a putative NLS (Kosugi *et al.* 2009) with multiple Thr-Pro and Ser-Pro motifs (Figure 4A) that are known to be phosphorylated (Costanzo *et al.* 2004; de Bruin *et al.* 2004). In addition, phosphorylation of Whi5 stimulates its exit from the nucleus, allowing Swi6 along with Swi4 and Mbp1 to stimulate transcription during G_1_/S (Costanzo *et al.* 2004; de Bruin *et al.* 2004). We wondered whether Ess1 might target phospho-Ser-Pro sites in Swi6 and Whi5 to stimulate their dephosphorylation, similar to the activity of Ess1 on the RNAPII CTD (Ma *et al.* 2012; Singh, *et al.* 2009; Werner-Allen *et al.* 2011). If Ess1 binds these sites and promotes dephosphorylation (by generating the preferred *cis*/*trans* isomer), then *ess1* catalytic mutants would be predicted to be defective for nuclear import of Swi6 and/or Whi5 proteins.

To test this idea, we used fluorescence microscopy to monitor nuclear import of Swi6- and Whi5-GFP fusion proteins in wild-type and *ess1^H164R^* mutant cells. SWI6_1-252_-GFP constructs driven by the *GAL1* promoter (Sidorova *et al.* 1995) were induced in galactose at 30° prior to visualization (Figure 4B, left panels). In wild-type cells, strong Swi6-GFP signals were detected in cell nuclei, mostly in the late M and G_1_ stages (large budded and unbudded cells, respectively), as expected (Harreman *et al.* 2004; Sidorova *et al.* 1995). In contrast, Swi6-GFP fluorescence in *ess1^H164R^* mutant cells was diffuse at all stages of the cell cycle, with signal in both the nucleus and the cytoplasm, indicative of a defect in nuclear localization. Note that cells were grown at the permissive temperature (30°) so failure to localize was not due to inviability or a measurable difference in the growth of *ess1^H164R^* mutant cells. The nuclear localization defects might have been more pronounced at the restrictive temperature (37°); however, interpretation would have been complicated due to nonspecific effects in dying cells.

One concern was that the defect in the localization of Swi6-GFP in *ess1^H164R^* mutant cells might have been a consequence of overexpression of the GFP-fusion proteins from the strong heterologous *GAL* promoter. To address this issue, the experiment was repeated in glucose-based medium using yeast strains bearing a chromosomally-integrated allele (*iSWI6-GFP*) under the control of the endogenous *SWI6* promoter. Similar results were obtained, with less prominent nuclear-localized Swi6-GFP and more cytoplasmic signal in the *ess1^H164R^* mutant cells compared with wild-type cells (Figure 4B, middle panels). Notably, detection was more challenging because of the lower expression level and the fact that the GFP construct was not fluorescence-enhanced. A visual scoring indicates showed Swi6-GFP signal in the nucleus in only 42% (n = 67) of *ess1^H164R^* mutant cells in G_2_ *vs.* 69% (n = 51) for the wild-type, and only 48% (n = 118) of *ess1^H164R^* mutant cells in M/G_1_ cells *vs.* 77%; (n = 101) for the wild type. Moreover, the intensity of nuclear staining was generally less in the mutant cells (Figure 4B and data not shown). Additional experiments using a centromeric plasmid expressing a full-length *SWI6-GFP* gene under control of the native *SWI6* promoter (Harreman *et al.* 2004) also showed a decrease in GFP nuclear signal in *ess1^H164R^* mutant cells relative to the wild-type (data not shown).

Similar experiments examined the localization of Whi5 in *ess1^H164R^* mutant cells. In this case, full-length *WHI5* fused to *GFP* was expressed from the *ADH1* promoter (Kosugi *et al.* 2009). In *ess1^H164R^* mutant cells, the fusion protein failed to localize to the nucleus and cytoplasmic staining was readily apparent (Figure 4B, right panels). We also examined the localization of GFP alone and found that localization was nonspecific (present throughout the cells), and more importantly that it was the same in both wild-type and *ess1^H164R^* mutant cells (Figure 4C, left panels). This control confirms that nuclear localization of the Swi6 and Whi5 GFP-fusion proteins (in wild-type cells) was not due to the GFP moiety. Finally, the ability of a control protein, GFP-Npl3, to localize to the nucleus in both wild-type and *ess1^H164R^* mutant cells indicates that the nuclear localization defects observed for Swi6-GFP and Whi5-GFP were not likely due to a general inability to localize proteins to the nucleus in *ess1^H164R^* cells.

Results of Western analysis using anti-GFP antibodies ruled out the possibility that lack of nuclear-localized Swi6-GFP or Whi5-GFP was due to failure to produce stable GFP-fusion proteins in *ess1^H164R^* mutant cells (Figure 4D). Levels of fusion proteins, expressed from the *GAL1* promoter, the native *SWI6* promoter, or an *ADH1* promoter (*WHI5*) were comparable in wild-type and *ess1^H164R^* cells. Taken together, these results indicate that Ess1 activity is required for nuclear localization of at least two cell cycle−dependent transcription regulators.

Previous work has shown nuclear functions for Ess1 in control of RNAP II (Singh *et al.* 2009). If Ess1 also plays a role in regulating the nuclear entry of Swi6, Whi5, and perhaps other proteins, it should also reside in the cytoplasm. This was tested by monitoring fluorescence in a strain in which *YFP* was integrated at the *ESS1* locus to express an Ess1-YFP fusion protein. Indeed, YFP fluorescence was observed in both nuclear and cytoplasmic compartments (Figure 4E), consistent with prior high-throughput localization studies (Huh *et al.* 2003). Localization of the Ess1(H164R) mutant protein fused to YFP was also both nuclear and cytoplasmic, although the fluorescence signal was reduced relative to the wild type (Figure 4E). This was not surprising given that Ess1(H164R) protein is less abundant, even at permissive temperature (Gemmill *et al.* 2005; Wu *et al.* 2000).

### Ess1 associates with Swi6 and Whi5 *in vivo*

**Figure 5:**
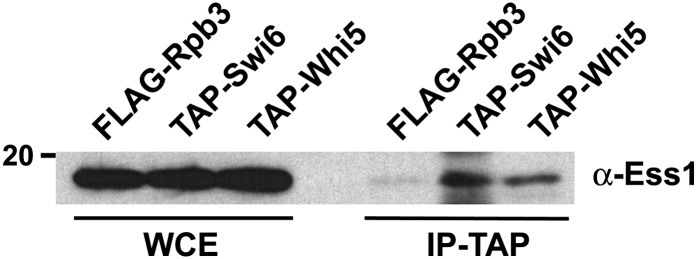
*In vivo* association of Ess1 with Swi6 and Whi5. Left lanes contain whole-cell extract (WCE) of strains expressing the indicated epitope-tagged proteins. Right lanes contain immunoprecipitates from strains expressing N-terminal tandem-affinity purification (TAP)-tagged versions of Swi6 and Whi5 expressed chromosomally from their normal promoters. Cells expressing FLAG-Rpb3 were used as a negative control. Immunoprecipitation of the TAP epitope (via the Protein A moiety) with IgG-sepharose beads was carried out using extract from the equivalent of 100 mL of cells at an OD_600_ of 1.4. Approximately one third of the precipitate (30 of 100 μL) was used per lane in SDS-PAGE before Western analysis. For the WCE, 1.25 μL from the total of 1 mL of input/WCE as used per lane. The faint band visible in the FLAG epitope tagged-Rpb3 IP might be due to cross reactivity of the IgG sepharose, since Ess1is known to be in a complex with Rpb1-Rpb3.

To determine whether Ess1 might target Swi6 and Whi5 in cells, we carried out CoIP/Western blot analysis using yeast strains expressing tandem-affinity purification (TAP)-tagged Swi6 or Whi5 proteins. The TAP-tagged proteins were pulled-down using IgG-sepharose beads, which binds the Protein A moiety within the tag. A control strain expressing a FLAG-epitope tagged protein (Rpb3 subunit of RNA pol II) also was included. The CoIP proteins were analyzed by Western blotting using polyclonal anti-Ess1 antibodies. Ess1 protein could be immunoprecipitated from strains expressing TAP-tagged Swi6 or Whi5 (Figure 5). Very little was precipitated from a negative control strain bearing a FLAG−epitope-tagged protein, although the residual amount might be due to nonspecific binding of Ess1 or FLAG-Rpb3 by the IgG beads (Ess1 is known to interact with the Rpb1-Rpb3 complex; Ma *et al.* 2012). In summary, these results indicate that Ess1 interacts with Swi6 and Whi5 in cell extracts. They do not, however, reveal whether this interaction is direct or mediated through other proteins.

### Ess1 interacts with the Swi6-NLS and Whi5-NLS *in vitro*

**Figure 6:**
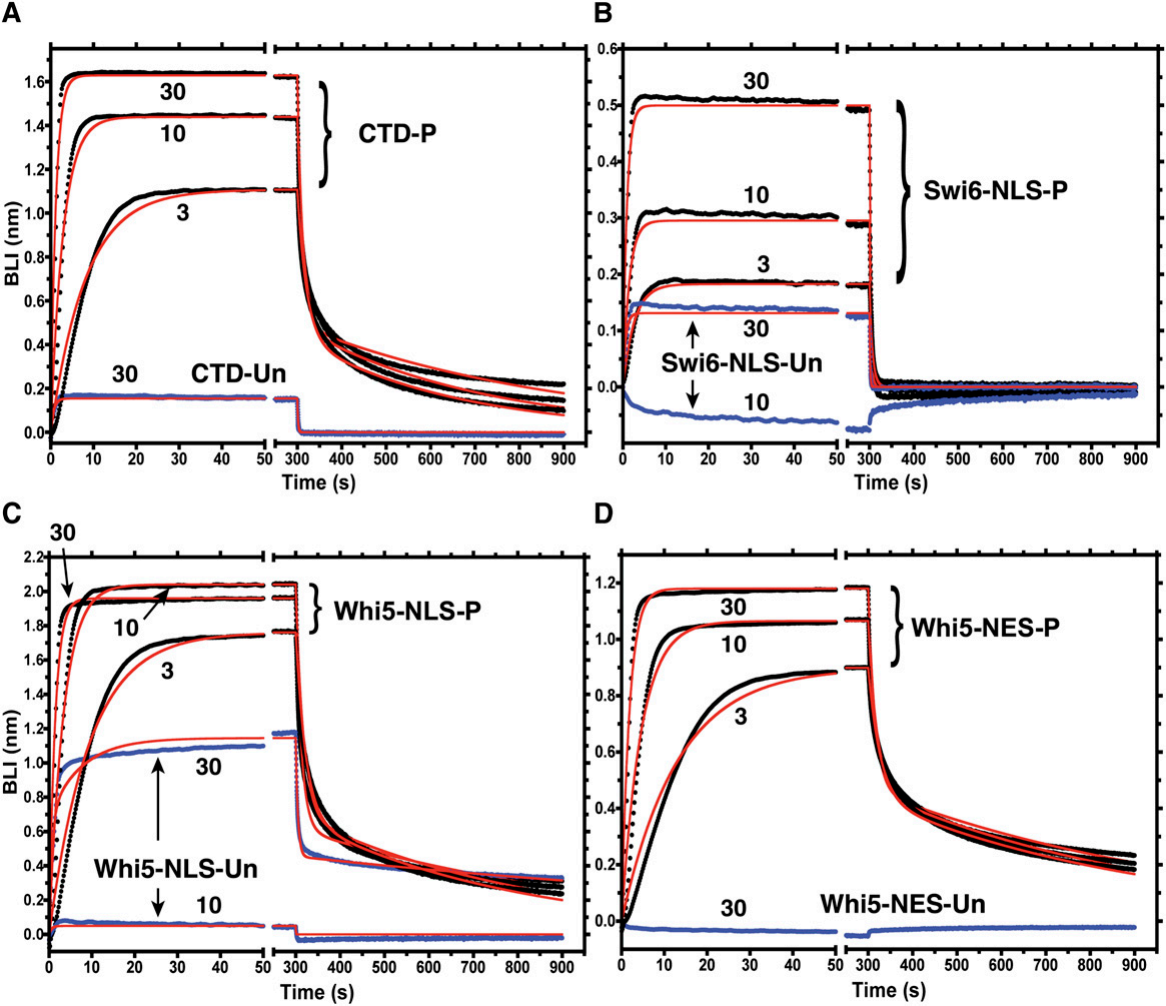
*In vitro* binding of Ess1 to Swi6 and Whi5 peptides. Kinetics for binding and dissociation of purified Ess1 protein with NLS and NES peptides were monitored using biolayer interferometry (BLI). Sensor-immobilized peptides: (A) carboxy-terminal domain (CTD) of RNA polymerase II (RNAPII), (B) Swi6-NLS, (C) Whi5-NLS, and (D) Whi5-NES. Data for phosphorylated peptides (P) are shown in black, unphosphorylated peptides (Un) in blue, and fitting curves are shown in red. Complete sequences of peptides are given in Table 5. Biotinylated peptides were loaded on individual streptavidin sensors, followed by a wash to remove unbound peptide. To measure Ess1 binding, sensors were transferred from buffer alone to buffer containing 3, 10, or 30 μM Ess1 protein (time zero) as indicated. After 300 sec, sensors were transferred to fresh buffer (without Ess1) to monitor Ess1 dissociation. Examples of raw data for these experiments, including the peptide-loading step and control sensors with no peptide are shown in Figure S1.

To determine whether Ess1 interacts directly with Swi6 and Whi5, we conducted binding assays by using purified Ess1 protein and phospho-Ser-Pro or Ser-Pro peptides corresponding to the NLS motif of Swi6 and both NLS and NES motifs of Whi5. Binding was assayed using BLI (Shah and Duncan 2013). In brief, biotinylated peptides corresponding to the NLS or NES motifs were immobilized on streptavidin-coated sensors and binding of purified Ess1 protein was measured using BLI. Ess1 protein binding to immobilized peptide changes the optical interference at the sensor surface, measured as nm of spectral shift. The peptides used are shown in Table 5. Using this approach, we monitored the kinetics of binding and dissociation of Ess1. As expected, Ess1 bound to a phosphorylated peptide corresponding to the CTD of RNAPII, whereas very little binding was observed when w used the cognate unphosphorylated CTD peptide (Figure 6A). Binding was dependent on Ess1 concentration (3, 10, and 30 μM were used). Examples of raw data obtained using BLI are shown in Figure S1.

**Table 5:** Peptides used for binding studies

Name	Sequence (Length)	Residues	*K*_appK_ (Kinetic), μM	*K*_appEq_ (Equilibrium), μM	*K*_app_ (Average), μM
CTD-P	GGSGGS**YSPTpSPSYS (15)**	CTD	2.58 ± 0.65	1.65 ± 0.06	2.12
CTD	GGSGGS**YSPTSPSYS (15)**	CTD	n.d.	n.d.	>300*^a^*
Swi6-NLS-P	GGSGGS**RELGpSPLKK (15)**	156−164	7.25 ± 4.63	10.08 ± 5.10	8.67
Swi6-NLS	GGSGGS**RELGSPLKK (15)**	156−164	n.d.	n.d.	>100*^a^*
Whi5-NLS-P	GGSG**GTPpSPPpSPPGI (15)**	56−66	2.87 ± 0.82	0.48 ± 0.46	1.67
Whi5-NLS	GGSG**GTPSPPSPPGI (15)**	56−66	n.d.	n.d.	>25
Whi5-NES-P	GGSGG**FLpSPpSPRLRpSPPT (18)**	152−164	3.25 ± 0.7	1.13 ± 0.18	2.19
Whi5-NES	GGSGG**FLSPSPRLRSPPT (18)**	152−164	n.d.	n.d.	>1500*^a^*

Amino acids in bold are derived from corresponding protein (residues numbers are indicated). The CTD sequence is based on the consensus heptapeptide repeat in the CTD of Rbp1. Values for K_appK_ and K_appEq_ for the phosphorylated peptides were determined as described in Figure S2. CTD, carboxy-terminal domain; P, phosphorylated peptide; n.d., not determined; NLS, nuclear localization sequences; NES, nuclear export signal; pS, phosphorylated serine.

a For the unphosphorylated peptides, *K*_appK_ and *K*_appEq_ could not be determined because binding was only significant at the greatest concentration of Ess1 (30 μM). Instead, *K*_app_ was estimated using a standard binding isotherm, F = E/(E + K_app_), where F is the fractional saturation of binding and is E is the concentration of Ess1. Assuming that unphosphorylated and phosphorylated peptides achieve the same signal for saturating binding of Ess1, F is calculated from the ratio U/*P*_sat_, where U is the binding signal of unphosphorylated peptide at 30 μM Ess1, and *P*_sat_ is the signal for saturated binding of the cognate phosphorylated peptide. The binding equation is then solved for *K*_app_.

Binding of Ess1 to the phosphorylated forms of the Swi6-NLS, Whi5-NLS, and Whi5-NES peptides was also observed (Figure 6, B−D). Binding to the Whi5 phospho-peptides was stronger than to the Swi6 phospho-peptide, more similar to binding of Ess1 to the phospho-CTD peptide (Figure 6 and Figure S2). Importantly, Ess1 binding was highly selective for the phosphorylated forms; the unphosphorylated peptides showed binding only marginally greater than background levels (no immobilized peptide; *e.g.*, Figure S1). To further demonstrate Ess1 binding specificity, a “nonsubstrate” peptide (H3 aa1-21) in either the phosphorylated form (Ser10-P) or the unphosphorylated form was tested. Ess1 did not bind to either form of the peptide (Figure S3). In fact, Ess1 binding, even to the phosphorylated H3 peptide was weaker than to the unphosphorylated CTD control peptide (Figure S3), and at the same level as a no-peptide control (data not shown). The strong preference of Ess1 for the phosphorylated forms of substrate-specific peptides is consistent with prior studies of both Ess1 and human Pin1 (Gemmill *et al.* 2005; Morris *et al.* 1999; Yaffe *et al.* 1997).

Kinetics of Ess1 dissociation could not be fit to a single exponential decay for the CTD or Whi5 phospho-peptides (not shown). For these peptides, dissociation appeared biphasic. The phospho-Swi6 peptide had a lower overall signal and faster decay, preventing the ability to distinguish between mono- and bi-phasic dissociation. Biphasic dissociation is consistent with the fact that Ess1 and other parvulin-class isomerases contain two phospho-Ser-Pro binding domains, a WW domain and a catalytic domain. However, we note that the biphasic dissociation curves could also result from re-binding of accumulating free Ess1 during dissociation from the immobilized substrates. Further experiments will be needed to distinguish between these possibilities. Due to the inability to resolve microscopic dissociation constants (*K*_D_), our estimates for apparent dissociation constants (*K*_app_) consider only the predominant, faster-dissociating signal.

*K*_app_ values for binding of Ess1 to the various peptides were estimated by the use of both kinetic and equilibrium binding data (Table 5). For Ess1 binding to the phosphorylated CTD peptide, the *K*_app_ was in the range of 2 μM, which is lower than the previous estimate of 60 μM from competition assays using fluorescence anisotropy (Gemmill *et al.* 2005). We are not certain what is responsible for this difference but we note that, based on the number of Ess1 protein molecules per cell (Gemmill *et al.* 2005), the intracellular concentration of Ess1 is estimated to be ~5 μM and, based on another study (Ghaemmaghami *et al.* 2003), the relative concentrations of Swi6 and Whi5 are within threefold of that of Ess1. The *K*_app_ values determined in the present study are in the range of these concentrations.

Among the peptide substrates tested, the relative strength of binding of Ess1 was: CTD-P ≈ Whi5-NLS-P ≈ Whi5-NES-P *>* Swi6NLS-*P* > >>> unphosphorylated peptides. The *K*_app_ for phosphorylated Whi5 peptides was ~2 μM, whereas the *K*_app_ for the phosphorylated Swi6 peptide was ~9 μM. This 4- to 5-fold reduction was not due to slower binding kinetics, as the on-rates (*k*_a_) or all the peptides, including the phosphorylated CTD peptide, were similar (1.75−2.71 × 10^4^ M^−1^s^−1^, Table S4). Instead, the dissociation rate (*k*_d_) for the Swi6 peptide (0.254 s^−1^) was ~3- to 5-fold faster than for the other peptides (Table S4). In summary, Ess1 binds directly and selectively to phosphorylated sequences *in vitro* that are targets of cyclin-dependent kinases *in vivo* and that direct nuclear localization.

## Discussion

### A model for Ess1 regulation of Swi6 and Whi5 nuclear localization

**Figure 7:**
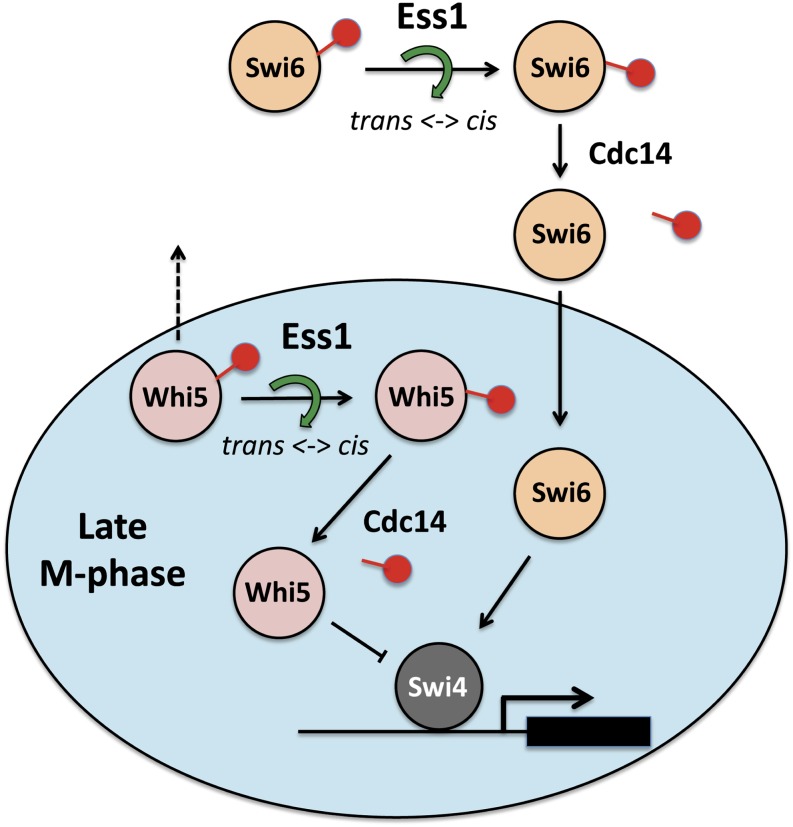
Model for regulation of Swi6 and Whi5 by Ess1-dependent isomerization. The model shows that Ess1 is required for correct cell cycle-dependent nuclear localization of Swi6 and Whi5. Ess1 may regulate nuclear import of Swi6 and Whi5 (during late M and early G_1_), and/or regulate nuclear retention of Swi6 and Whi5 during G_1_. Ess1 is proposed to bind phospho-Ser-Pro target sites within the nuclear localization sequences (NLS) of Swi6 and Whi5 and the nuclear export signal (NES) of Whi5 to induce *cis-trans* prolyl isomerization. This conformational switch may act directly to change nuclear import/retention (*e.g.*, via altered interaction with nuclear pore complexes) or it may act indirectly (as depicted in figure) to stimulate dephosphorylation of Swi6 and Whi5 by Cdc14. It is known that nuclear localization of Swi6 and Whi5 requires their dephosphorylation. The inability of Whi5 to enter the nucleus during late M could lead to premature exit from mitosis (*e.g.*, due to loss of Whi5-dependent repression of G_1_ cyclins) while the lack of nuclear Swi6 during G_1_ could prevent entry into S (*e.g.*, due to loss of Swi6 activation of G_1_ cyclins and S-phase enzymes) (not depicted in figure).

A model for Ess1 control of Swi6 and Whi5 localization consistent with our data are depicted in Figure 7. Phosphorylation of cyclin-dependent kinase sites within the NLS of Swi6 and Whi5 is known to keep Swi6 and Whi5 in the cytoplasm during S, G2 and early M phases of the cell cycle (Harreman *et al.* 2004; Sidorova *et al.* 1995). Phosphorylation of these sites blocks nuclear import of Swi6 and promotes nuclear export of Whi5 (Costanzo *et al.* 2004; Taberner *et al.* 2009; Wagner *et al.* 2009). In our model, phosphorylation would also promote binding of Ess1 to the phospho-Ser-Pro sites within the NLSs of Swi6 and Whi5, and the NES of Whi5. Ess1 binding and isomerization of the prolyl bonds within these localization sequences would generate conformational isomers (either *cis* or *trans*) that would render Swi6 and Whi5 competent for nuclear import and retention. In support of the model, we showed (1) that cells expressing a catalytically defective Ess1 prolyl isomerase have impaired nuclear localization of both Swi6 and Whi5 (Figure 4), (2) that Ess1 could be co-immunoprecipitated with Swi6 and Whi5 proteins (Figure 5), and (3) that purified Ess1 binds directly and preferentially to peptides representing the phosphorylated forms of Swi6 and Whi5 NLS/NES motifs (Figure 6). In addition, Swi6- and Whi5-regulated genes showed defects in induction in *ess1* mutant cells following cell cycle release, consistent with reduced nuclear import (Figure 3C).

There are several mechanisms by which Ess1 might regulate Swi6 and Whi6 nuclear localization. First, certain conformational isomers may be favored by the nuclear import machinery (or disfavored by the nuclear export machinery). That is, *cis* or *trans* isomers of Swi6 or Whi5 might show differential binding to the importins or exportins required for localization. A second, indirect mechanism, and one that we favor, would involve control of the phosphorylation state of Swi6 and Whi5 by Ess1 (as depicted in Figure 7). This mechanism is based on the known activity of Ess1 on the CTD of RNA polymerase II: Ess1 promotes CTD dephosphorylation at the pSer5-Pro6 site by converting the prolyl bond into the *cis* conformation which is favored by the Ssu72 phosphatase (Singh *et al.* 2009; Werner-Allen *et al.* 2011). Here, Ess1-catalyzed isomerization of the NLS/NES target sites in Swi6 and Whi5 would make them better substrates for the Cdc14 phosphatase, which has been shown to dephosphorylate these and other cell cycle regulators to stimulate their nuclear import and/or retention.

Cdc14 is primarily nuclear, and is released from the nucleolus during anaphase to promote mitotic exit (Geymonat *et al.* 2002; Jensen *et al.* 2002). Among its targets, Cdc14 dephosphorylates the NES of Whi5, thus preventing nuclear exit via the Msn5 karyopherin (Taberner *et al.* 2009). Cdc14 also dephosphorylates Ser160 in the NLS of Swi6 allowing nuclear accumulation, although it is still not clear how much of this effect is due to increased nuclear import *vs.* decreased nuclear export (Geymonat *et al.* 2004). Ess1, which is known to reside both in the nucleus and the cytoplasm (Arevalo-Rodriguez and Heitman 2005; Huh *et al.* 2003; and Figure 4E) could promote Cdc14 action in either or both compartments. In this way, Ess1 would promote resetting of the unphosphorylated (nuclear) state of cell-cycle regulators. In the absence of Ess1 activity, the action of Cdc14 would be inefficient due to the very slow rates of spontaneous isomerization of peptide-prolyl bonds. Further studies will be needed to determine whether, in fact, the Cdc14 phosphatase shows *cis*/*trans* selectivity for its activity on substrates.

### Genetic interactions and cell-cycle arrest phenotypes of *ess1* mutants

The aforementioned model might help explain some of the genetic interactions between Ess1 and several of the cell cycle transcription factors that we observed. For example, the synthetic lethality of *ess1^H164R^swi6*Δ double mutants might reflect additive effects of the absence of Swi6 protein (*swi6*Δ alone is not lethal) and mislocalization of Whi5 and other cell cycle regulators (due to the *ess1* mutation). Further genetic and biochemical studies will be needed to understand the genetic relationships identified in the SGA and growth assays of Figure 2. It is possible that mislocalization of Swi6, Whi5 or potentially other cell cycle regulators contribute to the mitotic arrest phenotype observed in *ess1* mutants (Lu *et al.* 1996; Wu *et al.* 2000). For example, the inability of Whi5 together with Swi6 to enter the nucleus in late M-phase might cause premature activation of genes they normally repress, leading to a precocious exit from M and mitotic catastrophe. Studies with *ess1* mutants have proved difficult because they do not follow classic cell-cycle arrest kinetics, as mutant cells can divide up to 7 times prior to arrest and nuclear fragmentation (Hanes *et al.* 1989). Moreover, isomerization of target proteins by Ess1 is not likely to be an all-or-none regulatory switch, but rather to contribute to the *efficiency* of regulation. Nonetheless, this study opens a new avenue for study of cell cycle regulation by prolyl isomerization in yeast.

### Binding of Ess1 to NLS

Before this study, the CTD of RNAPII large subunit was the only confirmed molecular target of Ess1 in yeast. In the present study, we found that Ess1 was present in complexes with Swi6 and Whi5 *in vivo* (Figure 5) and bound to the Swi6 and Whi5 NLS/NES peptides *in vitro* (Figure 6), suggesting that Swi6 and Whi5 are direct targets of Ess1. Binding was specific for the phosphorylated forms of the nuclear targeting sequences as expected given the specificity of Ess1 for phospho-Ser-Pro motifs. The greater affinity of Ess1 for Whi5-NLS and Whi5-NES peptides *vs.*. the Swi6-NLS peptides could be due to the fact that the Whi5 peptides contains multiple phospho-Ser-Pro motifs, or perhaps due to preferences for flanking sequence. The CTD sequence, despite having only a single phosphorylated Ser, bound Ess1 with an overall affinity comparable to that of the Whi5 peptides.

Although the Swi6 NLS used in our binding experiments has been demonstrated to be functional *in vivo* (Harreman *et al.* 2004), the Whi5 NLS peptide we used was based on a sequence predicted to have NLS acivity (Kosugi *et al.* 2009). A fragment of Whi5 (aa1-100) that contains this sequence (aa56-66) directs nuclear localization and the Ser-Pro sites within this sequence are phosphorylated *in vivo* (Taberner *et al.* 2009; Wagner *et al.* 2009), but it has not been demonstrated that this sequence is *sufficient* for regulated nuclear localization. By contrast, the Whi5-NES motif used in our experiments has been shown by mutational analysis (of the Ser-Pro sites) to be critical for nuclear retention *in vivo* (Taberner *et al.* 2009; Wagner *et al.* 2009).

Our *in vitro* binding studies provide estimates for the affinities of Ess1 for phosphorylated Ser-Pro-containing peptides of CTD, Swi6, and Whi5 that are in the low micromolar range (Table 5). For Pin1, it has been shown that the WW domain has >10-fold greater affinity for substrates than does the catalytic domain (Verdecia *et al.* 2000). Therefore, the majority of binding and dissociation measured in our experiments (Figure 6, Table 5) is probably due to the Ess1 WW domain. Additional binding experiments using individual Ess1 domains and/or mutant proteins will be needed to resolve the contribution of the PPIase catalytic domain. Moreover, the BLI binding assay we used should be applicable to the study of interactions between Ess1 and longer peptides or intact protein substrates that contain multiple Ser-Pro sites. This could reveal potential cooperative interactions due to simultaneous binding of the WW and catalytic domains to these substrates.

### Comparison with Pin1 in vertebrates

Pin1 binds a wide variety of signaling proteins to control their activity and/or protein−protein interactions (Liou *et al.* 2011). Perhaps the best studied cell-cycle target is the dual-specificity phosphatase, Cdc25, which dephosphorylates Cdc2/CyclinB to promote entry into mitosis. A large number of sites within Cdc25 are phosphorylated, and these are important for controlling its stability, cellular localization and activity (reviewed in Boutros *et al.* 2006; Karlsson-Rosenthal and Millar 2006). Pin1 binds specifically to pThr48-Pro49 and pThr67-Pro68 sites within the Cdc25C isoform (Zhou *et al.* 2000) and induces conformational changes (Stukenberg and Kirschner 2001), making them better substrates for the PP2A phosphatase, which prefers the *trans* conformation at these sites (Zhou *et al.* 2000). Although Pin1 appears to be important for Cdc25’s role in initiating mitosis, unlike Ess1 and Swi6/Whi5, it does not seem to regulate nuclear entry. Instead, Cdc25 nuclear entry is regulated by phosphorylation at non-proline-containing phospho-Ser sites, which are in turn bound by 14-3-3 proteins (Kumagai and Dunphy 1999; Yang *et al.* 1999).

It has been reported that Pin1 regulates nuclear localization of cyclin D1 (Liou *et al.* 2002). Pin1^−/−^ mouse embryo fibroblasts showed very little nuclear localized cyclin D1 compared with Pin1^+/+^ control cells. However, this result was compromised by the fact that levels of cyclin D1 protein were vastly reduced in these cells. So, although it is clear that Pin1 targets cyclin D1 and stabilizes the protein, whether it actually regulates nuclear import/export is not certain. A more convincing example is Pin1 regulation of nuclear localization of β-catenin (Ryo *et al.* 2001) which, along with its cell adhesion functions, transduces Wnt-pathway signals by translocating to the nucleus to stimulate gene transcription (Kim *et al.* 2013). Pin1 binds to pSer246-Pro247 in β-catenin and blocks its interaction with adenomatous polyposis coli protein (APC) (Ryo *et al.* 2001). Because APC helps shuttle β-catenin out of the nucleus (Henderson 2000), this allows nuclear accumulation of β-catenin. Whether Pin1 acts stoichiometrically to physically block β-catenin interaction with APC, or whether Pin1-induced isomerization of β-catenin prevents its interaction with APC was not investigated. In either case, Ess1 may work similarly to block export of Whi5 (or Swi6) from the nucleus, for example by blocking its interaction with the Msn5 exportin.

Finally, in a more recent study, Nakatsu *et al.* (2010) showed that Pin1 regulates nuclear localization of CRTC2, a CREB (cyclic AMP response element binding protein). Overexpression of Pin1 resulted in CRTC2 cytoplasmic localization, whereas Pin1 siRNA knockdown resulted in CRTC2 nuclear localization. Pin1 bound the NLS sequence of CRTC2 at pSer136-Pro137 and blocked its interaction with CREB. Although the mechanism by which Pin1 regulates CRTC2 nuclear localization was not determined, this study provides an example of regulation by a prolyl isomerase binding to an NLS. The results of our study of Ess1 in yeast provide a second example, and identify a tractable system in which to determine the molecular mechanisms.

## Supplementary Material

Supporting Information
