# Supplementary material for: The Yeast Ess1 Prolyl Isomerase Controls Swi6 and Whi5 Nuclear Localization
Source: G3 (Bethesda). 2014 Jan 27;4(3):523–37. doi: 10.1534/g3.113.008763 (PMC3962490; doi:10.1534/g3.113.008763)
Supplement: Supporting Information [file supp_4_3_523__index.html]

The Yeast Ess1 Prolyl Isomerase Controls Swi6 and Whi5 Nuclear Localization — Supporting Information 

# The Yeast Ess1 Prolyl Isomerase Controls Swi6 and Whi5 Nuclear Localization

## Supporting Information for Atencio *et al.*, 2014

**Files in this Data Supplement:**

- Supporting Information - Figures S1-S3 and Tables S1-S4 (PDF, 2 MB)
- Figure S1 - Representative BLI experiments. (PDF, 233 KB)
- Figure S2 - Estimation of apparent Kd values for binding of Ess1 to peptides. (PDF, 1 MB)
- Figure S3 - BLI experiment testing Ess1 binding to non-substrate a phospho-peptide. (PDF, 548 KB)
- Table S1 - Oligonucleotides used in this study. (PDF, 413 KB)
- Table S4 - Fitting Results for BLI Kinetic Assays (PDF, 303 KB)
- Table S2 - Complete list of SGA interactions with *ess1H164R* (.xls, 139 KB)
- Table S3 - Results of SlimMapper Gene Ontology (GO) analysis (.xls, 39 KB)
