# Supplementary material for: The Yeast Ess1 Prolyl Isomerase Controls Swi6 and Whi5 Nuclear Localization
Source: G3 (Bethesda). 2014 Jan 27;4(3):523–37. doi: 10.1534/g3.113.008763 (PMC3962490; doi:10.1534/g3.113.008763)
Supplement: Supporting Information [file supp_g3.113.008763_008763SI.pdf]

G3·Genes | Genomes | Genetics

# The yeast Ess1 prolyl isomerase controls Swi6 and Whi5 nuclear localization

David Atencio*, Cassandra Barnes*, Thomas M. Duncan*, Ian M. Willis§ and Steven D. Hanes*

*Department of Biochemistry and Molecular Biology, SUNY Upstate Medical University, Syracuse, NY 13210, §Department of Biochemistry, Albert Einstein College of Medicine, Bronx, NY 10461

Corresponding Author:
Steven D. Hanes, Ph.D.
Department of Biochemistry and Molecular Biology
SUNY Upstate Medical University
750 E. Adams St., Syracuse, NY 13210
haness@upstate.edu
(315) 464-9887

**DOI: 10.1534/g3.113.008763**

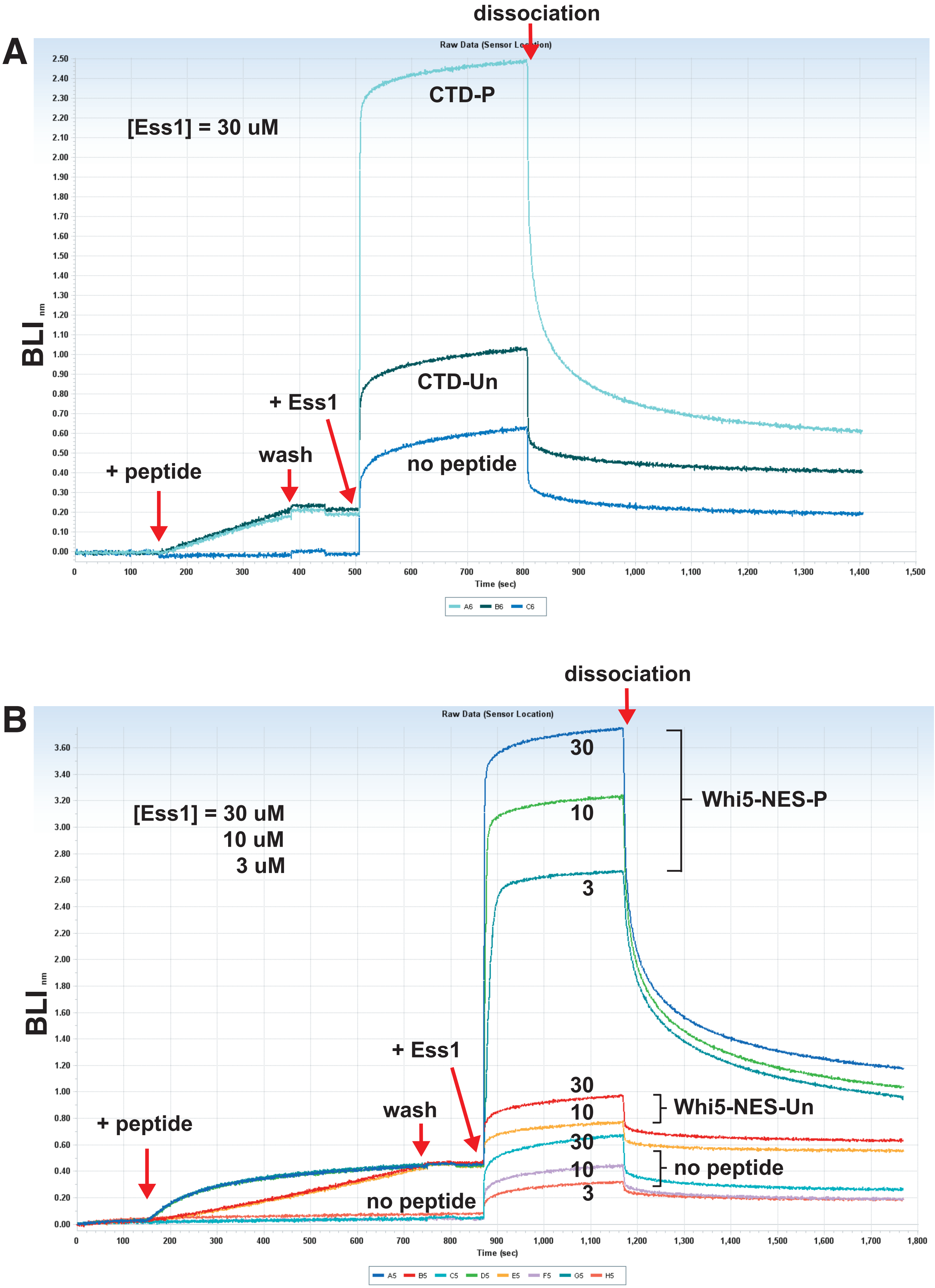
A
dissociation
Raw Data (Sensor Location)
[Ess1] = 30 uM
CTD-P
CTD-Un
no peptide
+ Ess1
wash
+ peptide
BLI nm
Time (sec)
A6 B6 C6
B
dissociation
Raw Data (Sensor Location)
[Ess1] = 30 uM
10 uM
3 uM
30
10
3
Whi5-NES-P
Whi5-NES-Un
no peptide
+ Ess1
wash
+ peptide
BLI nm
Time (sec)
A5 B5 C5 D5 E5 F5 G5 H5

**Figure S1 Representative BLI experiments.** (**A**) Raw data for binding of purified Ess1 protein (30 μM) to control peptides from RNA polymerase II CTD. Time of addition of biotinylated peptides (or no-peptide controls) to streptavidin-coated sensors is indicated by the arrow **(+ peptide**). After peptide binding, sensors were washed in buffer alone prior to transfer into buffer with Ess1 protein (**+Ess1**). Sensors with peptide and bound Ess1 were placed into fresh buffer (**dissociation**). (**B**) Example of raw data using peptides representing the nuclear export sequence (NES) of Whi5 at three concentrations of Ess1 as indicated. Samples A5, D5, and G5 are the Whi5-NES-P peptide with 30, 10, 3 μM Ess1 protein, respectively. Samples B5 and E5 are Whi5-NES-UN peptide with 30 and 10 μM Ess1 protein, respectively. Samples C5, F5, and H5 are "no-peptide-added" controls with 30, 10, 3 μM Ess1 protein, respectively. Note that the no-peptide controls show increased (background) signal with higher Ess1 protein concentrations, as expected**.** For both panels **A** and **B,** the (**P**) indicates phospho-Ser peptides, (**Un**) indicates unphosphorylated peptides. Full peptide sequences are given in the main text (**Table 5).**

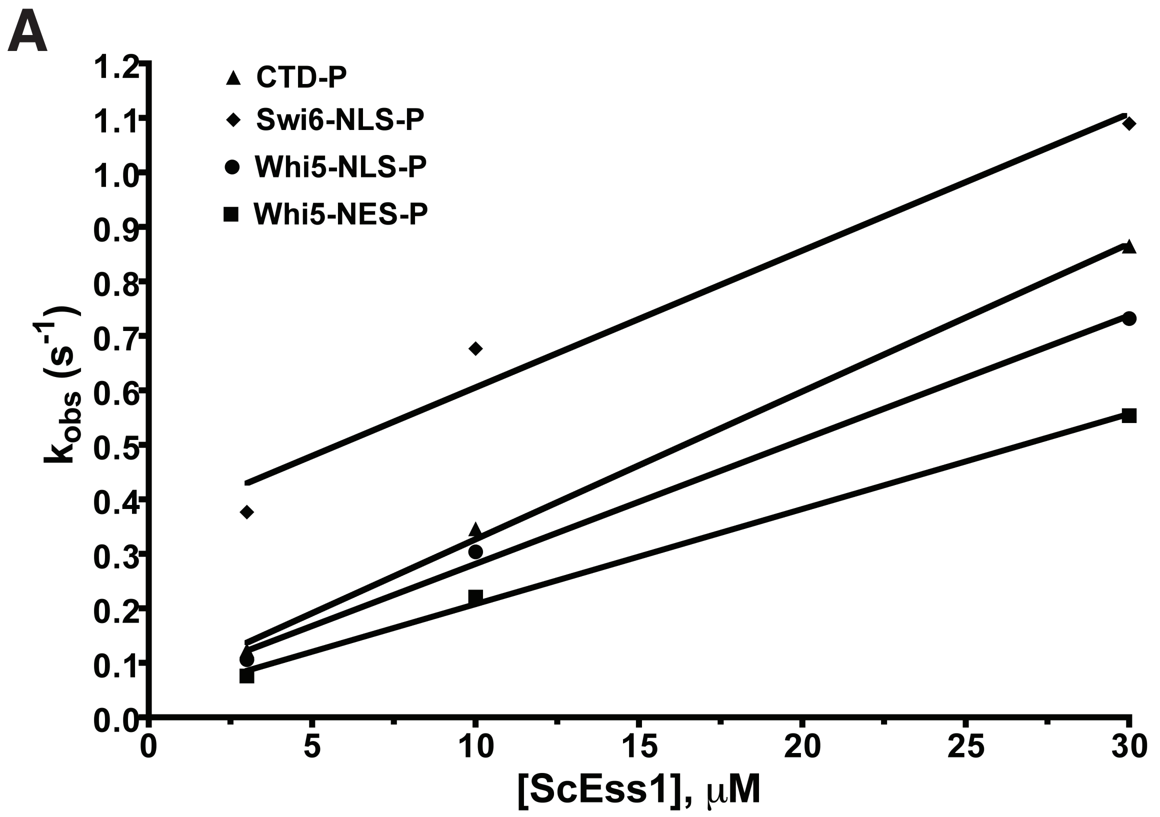
A
CTD-P
Swi6-NLS-P
Whi5-NLS-P
Whi5-NES-P
kobs (s-1)
1.2
1.1
1.0
0.9
0.8
0.7
0.6
0.5
0.4
0.3
0.2
0.1
0.0
0
5
10
15
20
25
30
[ScEss1], μM

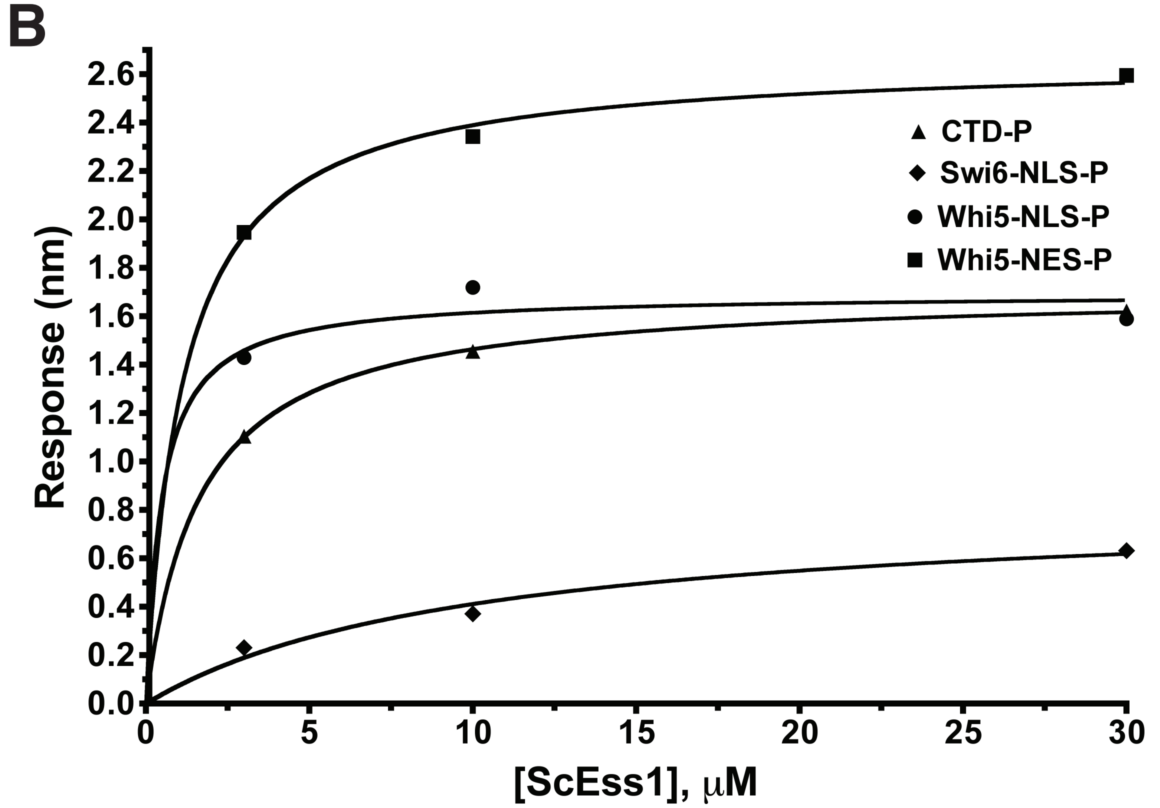
B
CTD-P
Swi6-NLS-P
Whi5-NLS-P
Whi5-NES-P
Response (nm)
2.6
2.4
2.2
2.0
1.8
1.6
1.4
1.2
1.0
0.8
0.6
0.4
0.2
0.0
0
5
10
15
20
25
30
[ScEss1], μM

**Figure S2 Estimation of apparent $K_d$ values for binding of Ess1 to peptides.** From the BLI kinetic assays of Ess1 binding with each peptide (Figure 6), regression analyses were used to determine the apparent dissociation constants ($K_{app}$) for binding of Ess1 to each of the query peptides. (A) For each phosphorylated peptide, observed rates ($k_{obs}$) for Ess1 association were plotted *vs.* the concentration of Ess1, and the slope provides an estimate of the 2$^{nd}$-order association rate ($k_a$, $M^{-1}s^{-1}$), which is listed in Table S4. The kinetic estimate for $K_D$ ($K_{appK}$, Table 5) was calculated as the ratio of $k_d/k_a$ values from Table S4. (B) The plateau value for binding at each Ess1 concentration (Figure 6) was assumed to represent the amount of Ess1/peptide complex at equilibrium. For each phosphorylated peptide, these equilibrium binding responses were plotted *vs.* Ess1 concentration and fit to a hyperbolic binding isotherm to obtain the equilibrium estimate for $K_D$ ($K_{appEq}$, Table 5).

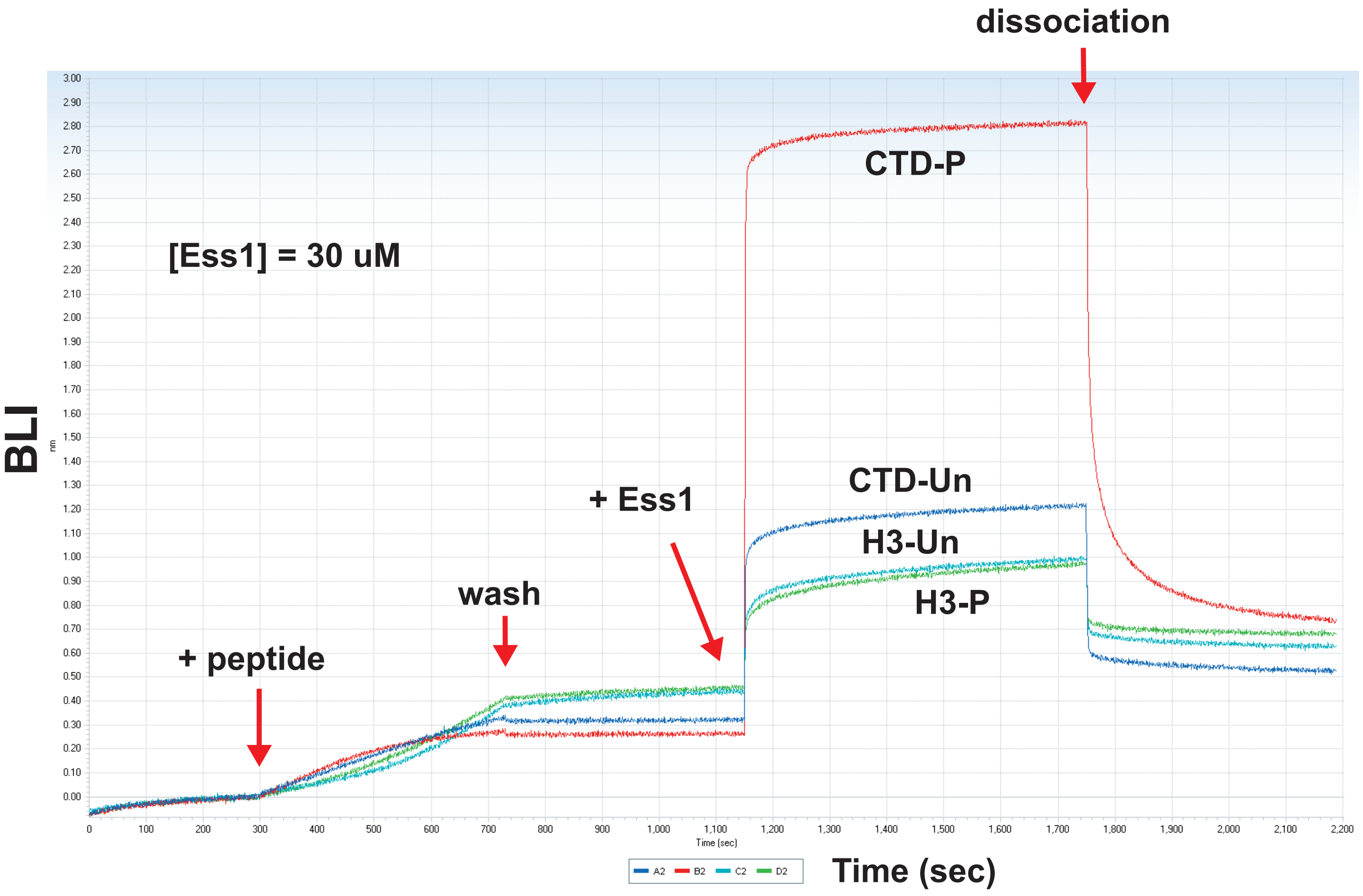


**Figure S3 BLI experiment testing Ess1 binding to non-substrate a phospho-peptide.** Raw data for binding of purified Ess1 protein (30 μM) to control peptides from the RNA polymerase II CTD (as in Figure S1), and to non-substrate control peptides from Histone H3 residues 1-21. **H3-Un** is unphosphorylated, **H3-P** is phosphorylated at serine 10. Experiment is carried out as described in Legend to Figure S1. Histone peptides were from Millipore (cat # 12-403; 12-427) The sequences are ARTKQTARK**S**TGGKAPRKQLA-GGK-Biotin, with the Ser residue that is phosphorylated in peptide H3-P highlighted. Levels of Ess1 binding to both H3 peptides is essentially identical to binding to no-peptide added control samples (data not shown).

**Table S1 Oligonucleotides used in this study**

| Oligo Name | Sequence | Use | Gene | reference |
|---|---|---|---|---|
| OW38 | CATAACCGACTACGGCAC | strain construction | ESS1 5'-F | Ma et al., 2012 |
| OW989 | TGTTTACAAAAAAATACAAGAATCCGTTACTAAAGATTCAGTATAGCGACCAGCATTCAC | strain construction | NAT-R | Ma et al., 2012 |
| OW1534 | TTCGCGAAGTAACCCTTCGTGGA | RT-qPCR control | SNR6-F | Ma et al., 2012 |
| OW1535 | GTAAAACGGTTCATCCTTATGCAG | RT-qPCR control | SNR6-R | Ma *et al.,* 2012 |
| UP007 | CAAGGTCACACTCCACTCCA | RT-qPCR | SWI4-F | This study |
| UP008 | GTAGTCCTGCTGACCCAAGC | RT-qPCR | SWI4-R | This study |
| UP009 | ACGACCGAGGTGAGTTATGG | RT-qPCR | SWI6-F | This study |
| UP010 | GCTTGCCTCTGGCAGTATTC | RT-qPCR | SWI6-R | This study |
| UP011 | GGATGGACGGACAAGACACT | RT-qPCR | WHI5-F | This study |
| UP012 | TCCACTTCGGTATCCGACTC | RT-qPCR | WHI5-R | This study |
| UP013 | CCACGTCCGTATCTTCCTCT | RT-qPCR | MBP1-F | This study |
| UP014 | ATATAGGGAGCGCTGTGTGG | RT-qPCR | MBP1-R | This study |
| UP015 | TTTACCCTCCGACGCTACTG | RT-qPCR | WHI3-F | This study |
| UP016 | CCTCCTCTTCTGCTGTTTGG | RT-qPCR | WHI3-R | This study |
| UP027 | TGACGATCCACTAGCGACTTC | RT-qPCR | CLN1-2-F | This study |
| UP028 | CTCGACCTGGCACCATTC | RT-qPCR | CLN1-2-R | This study |
| UP096 | ATTCGACTGCCTTGAGCTGT | RT-qPCR | CLB1-F | This study |
| UP097 | CCCATTCGTACTCCTCCAGA | RT-qPCR | CLB1-R | This study |
| UP094 | ACACCCAAGATGAGGCATTC | RT-qPCR | HCM1-F | This study |
| UP095 | GGGTGCAGAGGACTTTCTTG | RT-qPCR | HCM1-R | This study |
| UP098 | TTGCCTGTTCATTGCCTGTA | RT-qPCR | CLB6-F | This study |
| UP099 | GAATTTGGCCATGTTCCTTG | RT-qPCR | CLB6-R | This study |
| UP100 | CCCATCATTGCGATAAAAGGT | RT-qPCR | POL1-F | This study |
| UP101 | ACGCTTACCAAAAACGGAGA | RT-qPCR | POL1-R | This study |
| UP102 | TCAAGGAGGTCATGGAGGTC | RT-qPCR | SCJ1-F | This study |
| UP103 | CATCTGCAGAGCCACTACCA | RT-qPCR | SCJ1-R | This study |
| UP104 | GTCTATGAAGCAGCCCAAGC | RT-qPCR | HPR1-F | This study |
| UP105 | CATCCAACGCTTCCACTTTT | RT-qPCR | HPR1-R | This study |
| UP082 | TACATGGTCCATCGGCTGTA | RT-qPCR | CDC28-F | This study |
| P083 | TAGGTCTTTTCTGCGCCATT | RT-qPCR | CDC28-R | This study |
| P084 | TTTCTCCACCATTTGCCTTC | RT-qPCR | CLN3-F | This study |
| UP085 | TGAATTTTGTGGGATGCTCA | RT-qPCR | CLN3-R | This study |

| | | | | |
|---|---|---|---|---|
| UP086 | CCAACCTTCTACGGCGAATA | RT-qPCR | CDC14-F | This study |
| UP087 | TATCCTGCATGGATTCGTCA | RT-qPCR | CDC14-R | This study |
| UP088 | AGAAAAGAACGTCGGGACTG | RT-qPCR | NRM1-F | This study |
| UP089 | ATACCATCCTGGCATGAGGA | RT-qPCR | NRM1-R | This study |
| UP090 | GTTTTCAGCCGGAATATGGA | RT-qPCR | CLN2-F | This study |
| UP091 | CATTGGTTGCGTTATTGCTG | RT-qPCR | CLN2-R | This study |
| UP092 | CGAAGGATACCGCTATGGAA | RT-qPCR | HSL1-F | This study |
| UP093 | AGCATATGCGCATCATCAAA | RT-qPCR | HSL1-R | This study |
| UP108 | TATCGTTGAATCAGGAAGCGGTGTTCGTGTGATCAAGCGGGTAGGTGGTCG ACGGATCCCCGGG | strain construction | ess1$^{H164R}$ + YFP-HIS3 F | This study |
| OW318 | GGGAATAAGAACATAAAAAGGTGAGGTGGAGAAGCAAATGCCAACCATC GATGAATTCGAGCTCG | strain construction | ess1$^{H164R}$ + YFP-HIS3 R | This study |

F = forward; R = reverse

**Tables S2-S3**
Available for download at http://www.g3journal.org/lookup/suppl/doi:10.1534/g3.113.008763/-/DC1

**Table S2 Complete list of SGA interactions with *ess1*$^{H164R}$.** 429 genetic interactions were identified after setting a calibrated P-value of between +0.2 to +1.0 (aggravating interactions), or – 0.2 to -1.0 (alleviating interactions).

**Table S3 Results of SlimMapper Gene Ontology (GO) analysis.** List shows functional categories of genes that interact with *ESS1* derived from the SGA using a calibrated P-value cutoff value of 0.25. Analysis was done using SlimMapper http://www.yeastgenome.org/cgi-bin/GO/goSlimMapper.pl and yeast GO-Slim:Process was used to sort genes into indicated functional categories.

**Table S4 Fitting Results for BLI Kinetic Assays**

| Peptide | $k_a$ ($M^{-1}s^{-1}$) ±SD | $k_d$ ($s^{-1}$) ±SD | %Rmax ±SD |
|---|---|---|---|
| CTD-P | 27100 ±1220 | 0.0923 ±0.0274 | 71 ±1 |
| Swi6-NLS-P | 25100 ±4570 | 0.2537 ±0.0881 | 100 |
| Whi5-NLS-P | 22800 ±1420 | 0.0722 ±0.0384 | 72 ±1 |
| Whi5-NES-P | 17500 ±830 | 0.0503 ±0.0144 | 64 ±2 |

Summary of results for fitting curves of BLI kinetic data (**Fig. 6**). The dissociation rate ($k_d$) is an average (±standard deviation (SD)) for fits at 3 concentrations of each peptide (3, 10, 30 μM). With the exception of the Swi6-NLS-P peptide, dissociation appeared biphasic, and the largest portion of signal for bound Ess1 (%Rmax) dissociated with the $k_d$ value is shown; a smaller portion appeared to dissociate at a slower rate, which might have been due to re-binding as the concentration of free peptide increased. For Swi6-NLS-P, dissociation was faster and monophasic; all signal for bound Ess1 dissociated with the $k_d$ value shown. The $k_a$ value for each peptide (±SD) was obtained from the slope in supplemental **Fig. S2** (the dependence of association kinetics ($k_{obs}$) on the peptide concentration).
