# Supplementary material for: The Yeast Ess1 Prolyl Isomerase Controls Swi6 and Whi5 Nuclear Localization
Source: G3 (Bethesda). 2014 Jan 27;4(3):523–37. doi: 10.1534/g3.113.008763 (PMC3962490; doi:10.1534/g3.113.008763)
Supplement: Supporting Information [file supp_g3.113.008763_FigureS1.pdf]

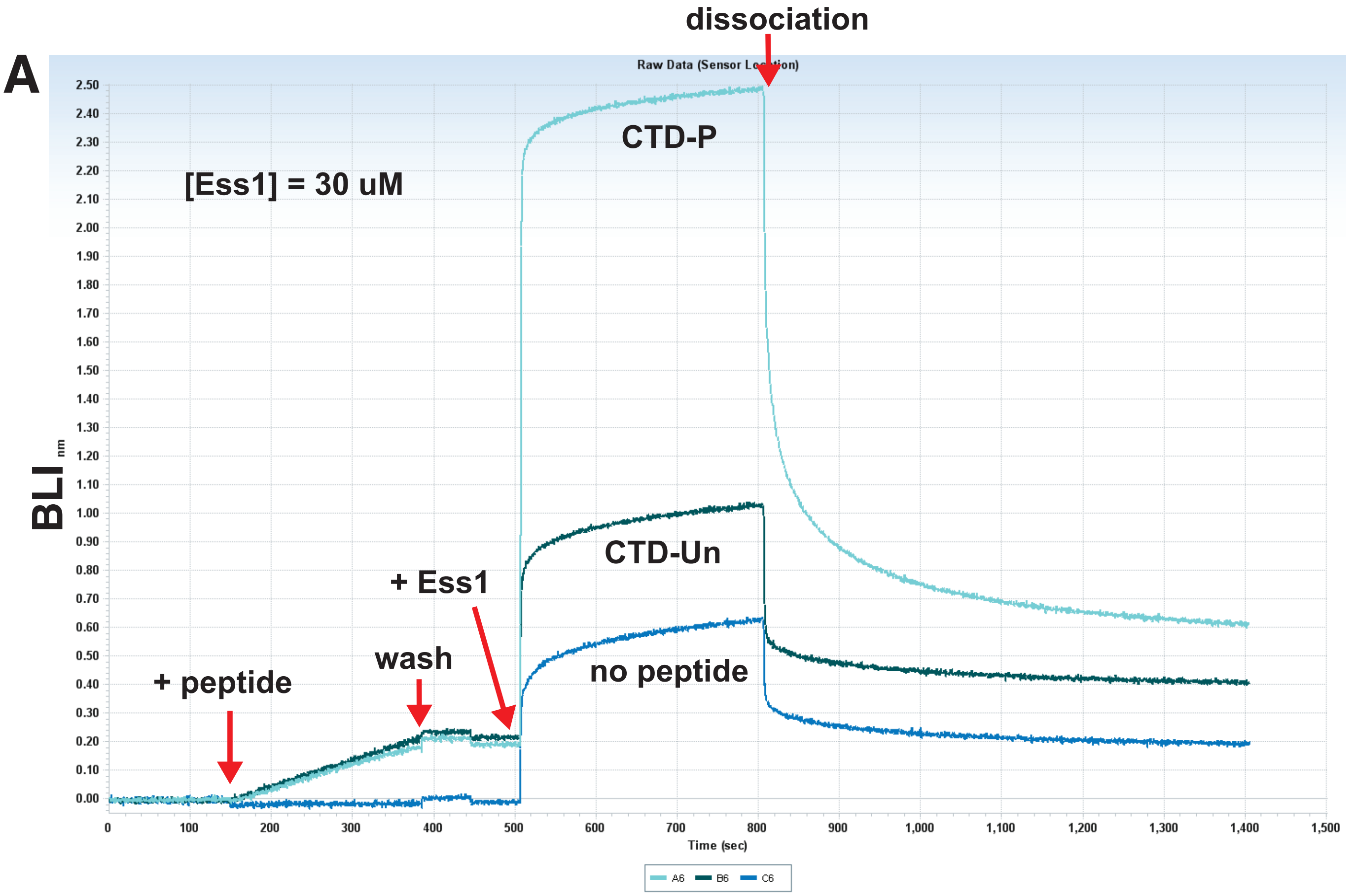
A
dissociation
Raw Data (Sensor Location)
[Ess1] = 30 uM
CTD-P
CTD-Un
no peptide
+ Ess1
wash
+ peptide
BLI nm
Time (sec)
A6 B6 C6

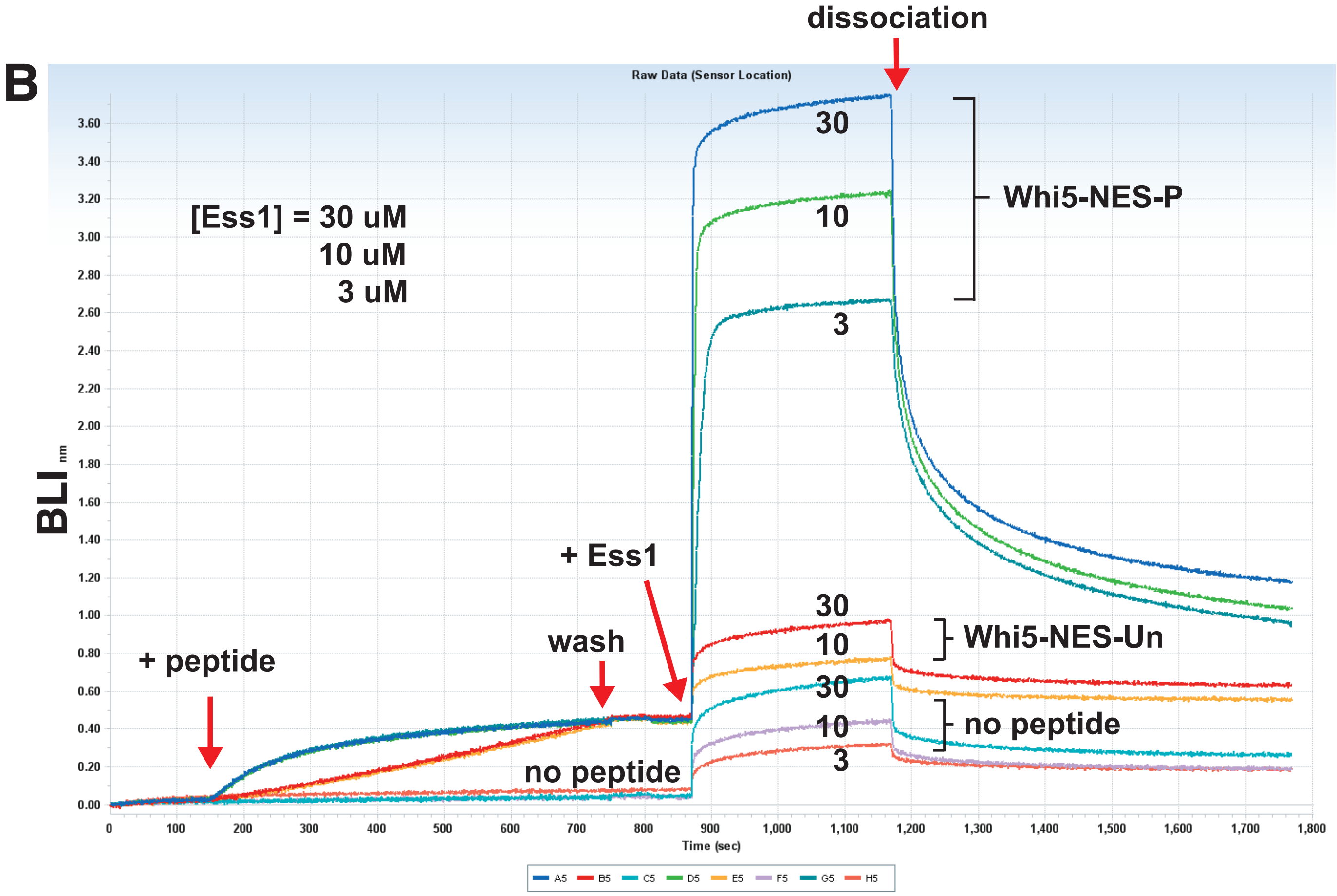
B
dissociation
Raw Data (Sensor Location)
[Ess1] = 30 uM
10 uM
3 uM
30
10
3
Whi5-NES-P
30
10
Whi5-NES-Un
30
10
3
no peptide
+ Ess1
wash
+ peptide
no peptide
BLI nm
Time (sec)
A5 B5 C5 D5 E5 F5 G5 H5

Figure S1 Representative BLI experiments. (**A**) Raw data for binding of purified Ess1 protein (30 μM) to control peptides from RNA polymerase II CTD. Time of addition of biotinylated peptides (or no-peptide controls) to streptavidin-coated sensors is indicated by the arrow **(+ peptide**). After peptide binding, sensors were washed in buffer alone prior to transfer into buffer with Ess1 protein (**+Ess1**). Sensors with peptide and bound Ess1 were placed into fresh buffer (**dissociation**). (**B**) Example of raw data using peptides representing the nuclear export sequence (NES) of Whi5 at three concentrations of Ess1 as indicated. Samples A5, D5, and G5 are the Whi5-NES-P peptide with 30, 10, 3 μM Ess1 protein, respectively. Samples B5 and E5 are Whi5-NES-UN peptide with 30 and 10 μM Ess1 protein, respectively. Samples C5, F5, and H5 are "no-peptide-added" controls with 30, 10, 3 μM Ess1 protein, respectively. Note that the no-peptide controls show increased (background) signal with higher Ess1 protein concentrations, as expected**.** For both panels **A** and **B,** the (**P**) indicates phospho-Ser peptides, (**Un**) indicates unphosphorylated peptides. Full peptide sequences are given in the main text (**Table 5).**
