# Supplementary material for: The Yeast Ess1 Prolyl Isomerase Controls Swi6 and Whi5 Nuclear Localization
Source: G3 (Bethesda). 2014 Jan 27;4(3):523–37. doi: 10.1534/g3.113.008763 (PMC3962490; doi:10.1534/g3.113.008763)
Supplement: Supporting Information [file supp_g3.113.008763_FigureS2.pdf]

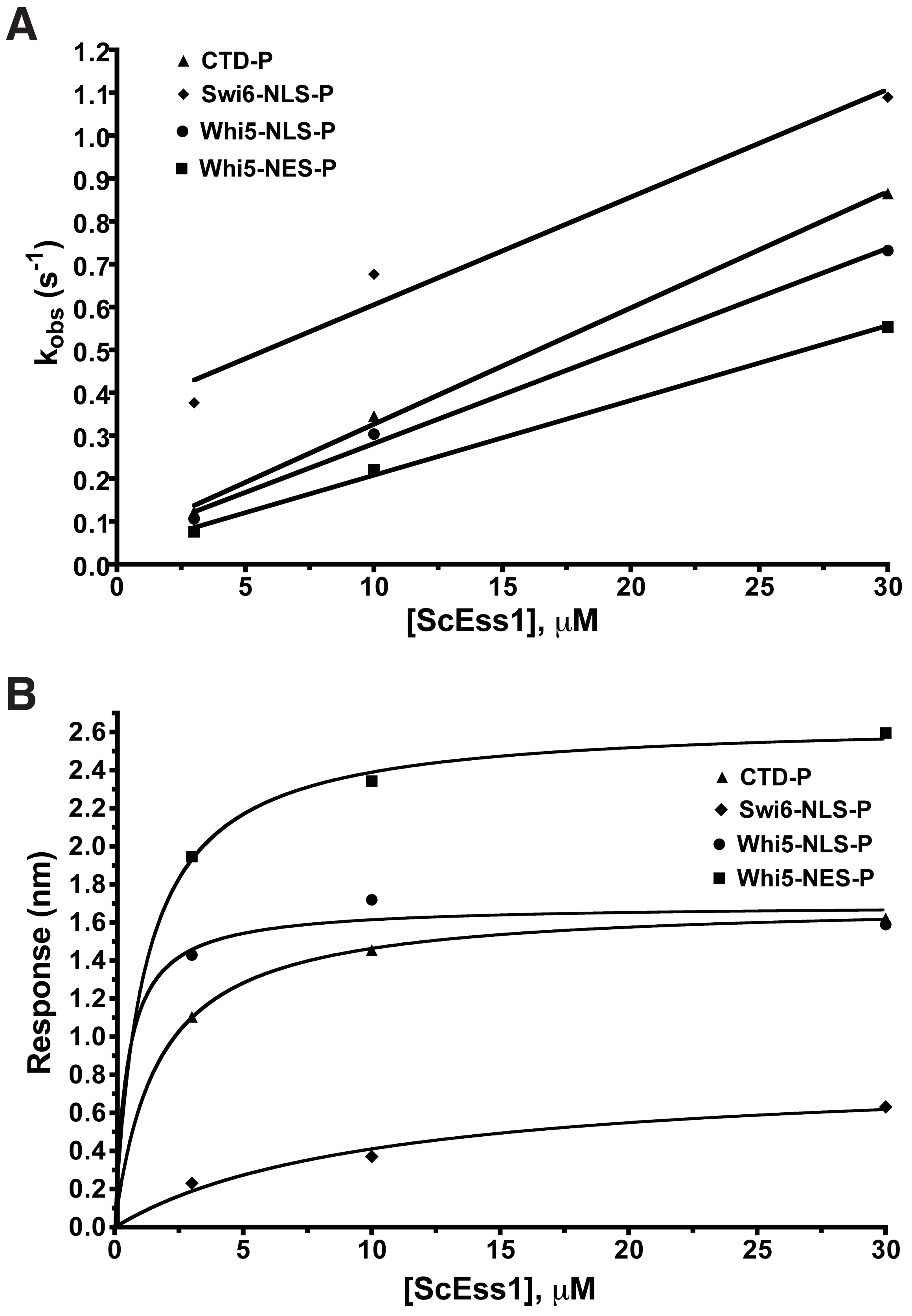
A
CTD-P
Swi6-NLS-P
Whi5-NLS-P
Whi5-NES-P
k_{obs} (s^{-1})
[ScEss1], μM
0.0
0.1
0.2
0.3
0.4
0.5
0.6
0.7
0.8
0.9
1.0
1.1
1.2
0
5
10
15
20
25
30
B
CTD-P
Swi6-NLS-P
Whi5-NLS-P
Whi5-NES-P
Response (nm)
[ScEss1], μM
0.0
0.2
0.4
0.6
0.8
1.0
1.2
1.4
1.6
1.8
2.0
2.2
2.4
2.6
0
5
10
15
20
25
30

Figure S2 Estimation of apparent K_d values for binding of Ess1 to peptides. From the BLI kinetic assays of Ess1 binding with each peptide (Figure 6), regression analyses were used to determine the apparent dissociation constants (K_{app}) for binding of Ess1 to each of the query peptides. (A) For each phosphorylated peptide, observed rates (k_{obs}) for Ess1 association were plotted *vs.* the concentration of Ess1, and the slope provides an estimate of the 2nd-order association rate (k_a, $M^{-1}s^{-1}$), which is listed in Table S4. The kinetic estimate for K_D (K_{appK}, Table 5) was calculated as the ratio of k_d/k_a values from Table S4. (B) The plateau value for binding at each Ess1 concentration (Figure 6) was assumed to represent the amount of Ess1/peptide complex at equilibrium. For each phosphorylated peptide, these equilibrium binding responses were plotted *vs.* Ess1 concentration and fit to a hyperbolic binding isotherm to obtain the equilibrium estimate for K_D (K_{appEq}, Table 5).
