# Supplementary material for: The Yeast Ess1 Prolyl Isomerase Controls Swi6 and Whi5 Nuclear Localization
Source: G3 (Bethesda). 2014 Jan 27;4(3):523–37. doi: 10.1534/g3.113.008763 (PMC3962490; doi:10.1534/g3.113.008763)
Supplement: Supporting Information [file supp_g3.113.008763_FigureS3.pdf]

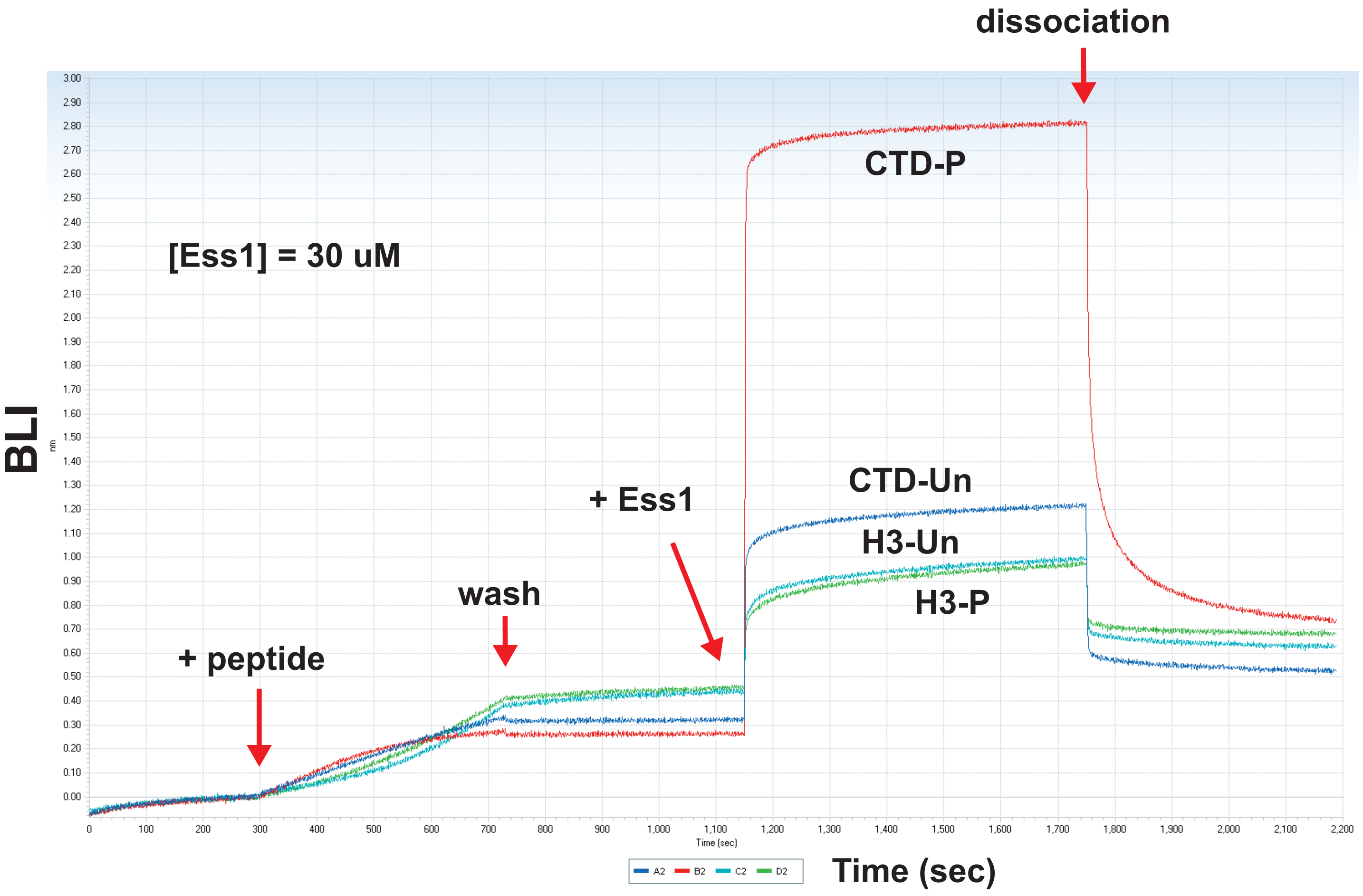

Figure S3 BLI experiment testing Ess1 binding to non-substrate a phospho-peptide. Raw data for binding of purified Ess1 protein (30 μM) to control peptides from the RNA polymerase II CTD (as in Figure S1), and to non-substrate control peptides from Histone H3 residues 1-21. **H3-Un** is unphosphorylated, **H3-P** is phosphorylated at serine 10. Experiment is carried out as described in Legend to Figure S1. Histone peptides were from Millipore (cat # 12-403; 12-427) The sequences are ARTKQTARK**S**TGGKAPRKQLA-GGK-Biotin, with the Ser residue that is phosphorylated in peptide H3-P highlighted. Levels of Ess1 binding to both H3 peptides is essentially identical to binding to no-peptide added control samples (data not shown).
