# Supplementary material for: The Yeast Ess1 Prolyl Isomerase Controls Swi6 and Whi5 Nuclear Localization
Source: G3 (Bethesda). 2014 Jan 27;4(3):523–37. doi: 10.1534/g3.113.008763 (PMC3962490; doi:10.1534/g3.113.008763)
Supplement: Supporting Information [file supp_g3.113.008763_TableS1.pdf]

Table S1 Oligonucleotides used in this study

Oligo Name	Sequence	Use	Gene	reference
OW38	CATAACCGACTACGGCAC	strain construction	ESS1 5’-F	Ma et al., 2012
OW989	TGTTTACAAAAAAATACAAGAATCCGTTACTAAAGATTCAGTATAGCGACCAGCATTCAC	strain construction	NAT-R	Ma et al., 2012
OW1534	TTCGCGAAGTAACCCTTCGTGGA	RT-qPCR control	SNR6-F	Ma et al., 2012
OW1535	GTAAAACGGTTCATCCTTATGCAG	RT-qPCR control	SNR6-R	Ma *et al.,* 2012
UP007	CAAGGTCACACTCCACTCCA	RT-qPCR	SWI4-F	This study
UP008	GTAGTCCTGCTGACCCAAGC	RT-qPCR	SWI4-R	This study
UP009	ACGACCGAGGTGAGTTATGG	RT-qPCR	SWI6-F	This study
UP010	GCTTGCCTCTGGCAGTATTC	RT-qPCR	SWI6-R	This study
UP011	GGATGGACGGACAAGACACT	RT-qPCR	WHI5-F	This study
UP012	TCCACTTCGGTATCCGACTC	RT-qPCR	WHI5-R	This study
UP013	CCACGTCCGTATCTTCCTCT	RT-qPCR	MBP1-F	This study
UP014	ATATAGGGAGCGCTGTGTGG	RT-qPCR	MBP1-R	This study
UP015	TTTACCCTCCGACGCTACTG	RT-qPCR	WHI3-F	This study
UP016	CCTCCTCTTCTGCTGTTTGG	RT-qPCR	WHI3-R	This study
UP027	TGACGATCCACTAGCGACTTC	RT-qPCR	CLN1-2-F	This study
UP028	CTCGACCTGGCACCATTC	RT-qPCR	CLN1-2-R	This study
UP096	ATTCGACTGCCTTGAGCTGT	RT-qPCR	CLB1-F	This study
UP097	CCCATTCGTACTCCTCCAGA	RT-qPCR	CLB1-R	This study
UP094	ACACCCAAGATGAGGCATTC	RT-qPCR	HCM1-F	This study
UP095	GGGTGCAGAGGACTTTCTTG	RT-qPCR	HCM1-R	This study
UP098	TTGCCTGTTCATTGCCTGTA	RT-qPCR	CLB6-F	This study
UP099	GAATTTGGCCATGTTCCTTG	RT-qPCR	CLB6-R	This study
UP100	CCCATCATTGCGATAAAAGGT	RT-qPCR	POL1-F	This study
UP101	ACGCTTACCAAAAACGGAGA	RT-qPCR	POL1-R	This study
UP102	TCAAGGAGGTCATGGAGGTC	RT-qPCR	SCJ1-F	This study
UP103	CATCTGCAGAGCCACTACCA	RT-qPCR	SCJ1-R	This study
UP104	GTCTATGAAGCAGCCCAAGC	RT-qPCR	HPR1-F	This study
UP105	CATCCAACGCTTCCACTTTT	RT-qPCR	HPR1-R	This study
UP082	TACATGGTCCATCGGCTGTA	RT-qPCR	CDC28-F	This study
P083	TAGGTCTTTTCTGCGCCATT	RT-qPCR	CDC28-R	This study
P084	TTTCTCCACCATTTGCCTTC	RT-qPCR	CLN3-F	This study
UP085	TGAATTTTGTGGGATGCTCA	RT-qPCR	CLN3-R	This study

UP086	CCAACCTTCTACGGCGAATA	RT-qPCR	CDC14-F	This study
UP087	TATCCTGCATGGATTCGTCA	RT-qPCR	CDC14-R	This study
UP088	AGAAAAGAACGTCGGGACTG	RT-qPCR	NRM1-F	This study
UP089	ATACCATCCTGGCATGAGGA	RT-qPCR	NRM1-R	This study
UP090	GTTTTCAGCCGGAATATGGA	RT-qPCR	CLN2-F	This study
UP091	CATTGGTTGCGTTATTGCTG	RT-qPCR	CLN2-R	This study
UP092	CGAAGGATACCGCTATGGAA	RT-qPCR	HSL1-F	This study
UP093	AGCATATGCGCATCATCAAA	RT-qPCR	HSL1-R	This study
UP108	TATCGTTGAATCAGGAAGCGGTGTTCGTGTGATCAAGCGGGTAGGTGGTCG ACGGATCCCCGGG	strain construction	$ess1^{H164R}$ + YFP-HIS3 F	This study
OW318	GGGAATAAGAACATAAAAAGGTGAGGTGGAGAAGCAAATGCCAACCATC GATGAATTCGAGCTCG	strain construction	$ess1^{H164R}$ + YFP-HIS3 R	This study

F = forward; R = reverse

Tables S2-S3
Available for download at http://www.g3journal.org/lookup/suppl/doi:10.1534/g3.113.008763/-/DC1

Table S2 Complete list of SGA interactions with *ess1*^{H164R}. 429 genetic interactions were identified after setting a calibrated P-value of between +0.2 to +1.0 (aggravating interactions), or – 0.2 to -1.0 (alleviating interactions).

Table S3 Results of SlimMapper Gene Ontology (GO) analysis. List shows functional categories of genes that interact with *ESS1* derived from the SGA using a calibrated P-value cutoff value of 0.25. Analysis was done using SlimMapper http://www.yeastgenome.org/cgi-bin/GO/goSlimMapper.pl and yeast GO-Slim:Process was used to sort genes into indicated functional categories.
