# Supplementary material for: The Yeast Ess1 Prolyl Isomerase Controls Swi6 and Whi5 Nuclear Localization
Source: G3 (Bethesda). 2014 Jan 27;4(3):523–37. doi: 10.1534/g3.113.008763 (PMC3962490; doi:10.1534/g3.113.008763)
Supplement: Supporting Information [file supp_g3.113.008763_TableS4.pdf]

Table S4 Fitting Results for BLI Kinetic Assays

Peptide	k_a ($M^{-1}s^{-1}$) ±SD	k_d (s^{-1}) ±SD	%Rmax ±SD
CTD-P	27100 ±1220	0.0923 ±0.0274	71 ±1
Swi6-NLS-P	25100 ±4570	0.2537 ±0.0881	100
Whi5-NLS-P	22800 ±1420	0.0722 ±0.0384	72 ±1
Whi5-NES-P	17500 ±830	0.0503 ±0.0144	64 ±2

Summary of results for fitting curves of BLI kinetic data (**Fig. 6**). The dissociation rate (k_d) is an average (±standard deviation (SD)) for fits at 3 concentrations of each peptide (3, 10, 30 μM). With the exception of the Swi6-NLS-P peptide, dissociation appeared biphasic, and the largest portion of signal for bound Ess1 (%Rmax) dissociated with the k_d value is shown; a smaller portion appeared to dissociate at a slower rate, which might have been due to re-binding as the concentration of free peptide increased. For Swi6-NLS-P, dissociation was faster and monophasic; all signal for bound Ess1 dissociated with the k_d value shown. The k_a value for each peptide (±SD) was obtained from the slope in supplemental **Fig. S2** (the dependence of association kinetics (k_{obs}) on the peptide concentration).
